# Neurophysiological correlates of ketamine-induced dissociative state in bipolar disorder: insights from real-world clinical settings

**DOI:** 10.1038/s41380-025-02889-2

**Published:** 2025-01-14

**Authors:** Claudio Agnorelli, Alessandra Cinti, Giovanni Barillà, Francesco Lomi, Adriano Scoccia, Alberto Benelli, Francesco Neri, Carmelo Luca Smeralda, Alessandro Cuomo, Emiliano Santarnecchi, Elisa Tatti, Kate Godfrey, Francesca Tarantino, Andrea Fagiolini, Simone Rossi

**Affiliations:** 1 Department of Molecular Medicine, Division of Psychiatry, School of Medicine, University of Siena, Siena, Italy; 2 Siena Brain Investigation and Neuromodulation Lab, Department of Medicine, Surgery and Neuroscience, University of Siena, Siena, Italy; 3 Precision Neuroscience & Neuromodulation Program, Gordon Center for Medical Imaging, Department of Radiology, Neurology, Psychiatry, Massachusetts General Hospital, Harvard Medical School, Boston, MA USA; 4 Department of Molecular, Cellular & Biomedical Sciences, CUNY, School of Medicine, New York, NY USA; 5 Centre for Psychedelic Research, Division of Psychiatry, Department of Brain Sciences, Imperial College London, London, UK; 6 Unit of Anesthesia and Neurological Intensive Care, Department of Neurological and Motor Sciences, University of Siena, Siena, Italy; 7 Present Address: Centre for Psychedelic Research, Division of Psychiatry, Department of Brain Sciences, Imperial College London, London, UK

**Keywords:** Neuroscience, Predictive markers, Bipolar disorder

## Abstract

Ketamine, a dissociative compound, shows promise in treating mood disorders, including treatment-resistant depression (TRD) and bipolar disorder (BD). Despite its therapeutic potential, the neurophysiological mechanisms underlying ketamine’s effects are not fully understood. This study explored acute neurophysiological changes induced by subanesthetic doses of ketamine in BD patients with depression using electroencephalography (EEG) biomarkers. A cohort of 30 BD (F = 12) inpatients with TRD undergoing ketamine treatment was included in the study. EEG recordings were performed during one of the ketamine infusions with doses ranging from 0.5 to 1 mg/kg, and subjective effects were evaluated using the Clinician-Administered Dissociative States Scale (CADSS). Both rhythmic and arrhythmic features were extrapolated from the EEG signal. Patients who exhibited a clinical response to ketamine treatment within one week were classified as early responders (ER), whereas those who responded later were categorized as late responders (LR). Ketamine reduced low-frequency spectral power density while increasing gamma oscillatory power. Additionally, ketamine flattened the slope of the power spectra, indicating altered scale-free dynamics. Ketamine also increased brain signal entropy, particularly in high-frequency bands. Notably, LR exhibited greater EEG changes compared to ER, suggesting endophenotypic differences in treatment sensitivity. These findings provide valuable insights into the neurophysiological effects of ketamine in BD depression, highlighting the utility of EEG biomarkers for assessing ketamine’s therapeutic mechanisms in real-world clinical settings. Understanding the neural correlates of ketamine response may contribute to personalized treatment approaches and improved management of mood disorders.

## Introduction

Ketamine, a versatile compound with various applications spanning anesthesia, antidepressant treatment, recreational use and abuse, has recently taken center stage in neuropsychiatric research. Several clinical trials have validated ketamine’s therapeutic properties in a variety of psychiatric conditions, demonstrating high efficacy against treatment-resistant depression (TRD), especially for suicidal ideation, and promising results for bipolar disorder (BD), post-traumatic stress disorder, and substance use disorders [1–6]. This bears huge importance given the growing prevalence of mood disorders and the limitations of conventional antidepressants [7]. At subanesthetic doses, ketamine triggers an altered state of consciousness that some authors describe as dissociation, similar to symptoms seen in dissociative disorders [8], while others liken it to a psychedelic experience [9, 10]. In the hour following ketamine’s administration as a treatment for depression, patients commonly report unusual bodily sensations, a sense of peace, disinhibition, and altered perception [11]. A systematic review investigating the link between ketamine-induced subjective effects and antidepressant response produced mixed findings. Of all the studies analyzed, only three reported a significant correlation between antidepressant response and scores on the Clinician Administered Dissociative States Scale (CADSS) or the Brief Psychotic Rating Scale (BPRS) [12]. Using the altered states of consciousness questionnaire (ASCQ), one study demonstrated that a stronger antidepressant response to ketamine was associated with experiences of unity, spirituality, and insight [13], though another study found that non-responders scored higher on dread of ego dissolution [14]. In patients with substance-use disorder, the mystical-type effects of ketamine, measured via the Hood Mysticism Scale (HMS), were found to mediate the reduction in patient’s cocaine use and craving [6].

Indeed, the subjective effects of ketamine appear to be highly variable, influenced significantly by factors such as dosage, individual differences, the setting of administration, and the instrument employed to assess the experience. This variability underscores the need to identify reliable biomarkers of acute neurophenomenology of ketamine in diverse patient populations and contexts.

Electroencephalography (EEG) is one of the most appropriate neuroimaging method to flexibly investigate the neural dynamics of psychoactive drugs due to its high temporal resolution and non-invasiveness. The neural signal recorded by EEG displays a diverse combination of rhythmic and arrhythmic patterns [15]. Rhythmic patterns emerge from oscillatory network activity with a characteristic time scale [16], while arrhythmic patterns lack confinement to any specific scale, reflecting nonlinear dynamics [17]. In both healthy subjects and patients with depression, a single continuous infusion of a subanesthetic dose of ketamine modulates brain rhythmic activity by decreasing spectral power in the low frequencies, such as δ [18–24] and θ [20, 22, 24, 25], and also in the α [18–24] and β [23, 25–29] bands, while increasing γ [20, 22–27, 29] frequency.

In one study, the reduction of α power induced by ketamine was found to correlate with depersonalization scores of the CADSS [20], and with alterations of elementary imagery of the ASCQ by another [22]. In patients with TRD, frequency-specific EEG changes induced by ketamine were predictive of decreases in depressive symptoms (i.e., θ, α, γ) and suicidal ideation (i.e., α) [30, 31], but not consistently across studies [32]. In recent years, the arrhythmic and non-linear dynamics of the neural signal have progressively been studied and characterized [33]. One such property is the scale-free (also termed fractal) activity, which adheres to a 1/f power-law relationship, expressing the property of brain signal to show an inverse relationship between power and frequency [16]. While the physiological mechanisms by which power-law scaling is generated in the brain are poorly understood and their significance remains controversial [15], the potential functional importance of power-law scaling in the brain is underscored by its alteration in various neuropsychiatric conditions [34–36]. Muthukumaraswamy and Liley demonstrated that the Power Law Exponent (PLE) of the brain signal recorded at rest is sensitive to various pharmacological interventions, including ketamine. In particular, a subanesthetic dose of ketamine was found to decrease the PLE exponent at frequencies between 5 and 100 Hz in healthy subjects [37]. To date, the effect of ketamine on PLE and its relationship with the subjective effects of the drug and therapeutic response in patients has not been investigated. In addition to scale-free properties, the high temporal resolution provided by EEG signal makes it ideal for determining measures of complexity and entropy of brain activity. One such measure is the Lempel-Ziv complexity (LZc), which assesses the level of compressibility and diversity of a signal [38]. Ketamine was observed to consistently increase spontaneous brain complexity [38–41], with one study reporting a correlation with the intensity of the subjective experience [38]. To date, there is very limited research on the characterization of complexity in patient populations and its association with therapeutic response, with only one investigation in a cohort of late-life TRD patients [42].

In summary, while some evidence suggests the potential utility of EEG metrics as biomarkers for ketamine-induced altered states of consciousness and therapeutic effects, further investigation is required. Data concerning the impact of ketamine on non-linear brain dynamics, their interaction with rhythmic neural activity, and their phenomenological and clinical implications, is currently lacking. Critically, the escalating use of ketamine within psychiatry raises the crucial issue of identifying robust biomarkers of ketamine’s mechanism of action [43]. Moreover, limited evidence exists regarding ketamine response in complex patient populations with diverse demographics and often undergoing poly-pharmacological treatments in real-world hospital settings. Large-scale studies and clinical observations have consistently highlighted significant variability in patients’ neural therapeutic response to ketamine and its brain-based correlates, with response rates oscillating between 35 and 60% [44]. Importantly, while some patients experience rapid antidepressant effects after a single infusion, a substantial proportion of individuals require multiple infusions to achieve a clinically meaningful response [45, 46].

In this study, we employed a portable 32-channel EEG headset to 1) characterize the neurophysiological underpinnings of the dissociative state induced by ketamine in BD patients in a clinical, real-world, setting, and to 2) explore the relationship between these neurophysiological markers, the dissociative subjective effects, and the treatment response to ketamine. Based on real-world observations of ketamine therapy, we hypothesized that endophenotypic differences in therapeutic response would correspond to distinct neurophysiological patterns. However, given the lack of solid evidence in this area, we adopted a data-driven approach to analyze the data in an unbiased manner.

## Materials and methods

### Patient population

The study included 30 patients with BD (both type 1 and 2) currently undergoing a major depressive episode requiring hospitalization (Table 1). The severity of depression at inclusion was assessed with the Montgomery-Åsberg Depression Rating Scale (MADRS) and a semi-structured interview conducted by a licensed psychiatrist. Acute suicidal ideation, presence of psychotic symptoms, current dependence on alcohol and other substances of abuse, and presence of other severe medical conditions were grounds for exclusion. All patients included in the study were undergoing concomitant poly-psychotropic treatment during the study (Table 1). Patients were required to provide informed consent to be included in the study. The study was approved by the research ethics committee of the University Hospital of Siena and all procedures were performed in accordance with the institution’s guidelines and regulations.

**Table 1:** Demographics, drugs, and psychometric measurements.

	TRD-BD (*N* = 30)	ER (*N* = 18)	LR (*N* = 12)	*p* value
Sex	M = 18, F = 12	M = 11, F = 7	M = 7, F = 5	0.901
Age	51 ± 13	52 ± 11	48 ± 15	0.671
Education	15.4 ± 3.6	16 ± 3.4	14.5 ± 3.8	0.300
SSRI (T0)	77%	78%	75%	0.885
SNRI (T0)	17%	11%	25%	0.342
Lithium (T0)	80%	72%	92%	0.211
Valproate (T0)	43%	50%	33%	0.388
Antipsychotics (T0)	73%	72%	75%	0.677
Antiepileptics (T0)	47%	50%	42%	0.788
Benzodiazepines (T0)	47%	44%	50%	0.890
Bupropion (T0)	13%	17%	8%	0.541
Other medications (T0)	43%	39%	50%	0.571
MADRS (T0)	38 ± 5	38 ± 5	38 ± 4	0.815
MADRS (T1)	21 ± 6	17 ± 3	27 ± 5	<0.001*
MADRS (T2)	13 ± 3	14 ± 3	12 ± 4	0.242
Ketamine dose mg/kg (T2)	0.7 ± 0.2	0.6 ± 0.1	0.9 ± 0.09	<0.001*
CADSS Tot (T2)	26 ± 15	29 ± 17	22 ± 13	0.253
CADSS Depersonalization (T2)	8 ± 3	9 ± 5	7 ± 5	0.100
CADSS Derealization (T2)	14 ± 8	15 ± 8	12 ± 6	0.218
CADSS Amnesia (T2)	2 ± 2	3 ± 2	1 ± 1	0.261
N° of previous ketamine administrations (T0-T2)	9 ± 8	7 ± 8	11 ± 7	0.006*

*Mann–Whitney U test with α-level = 0.05.

### Study design

**Fig. 1:**
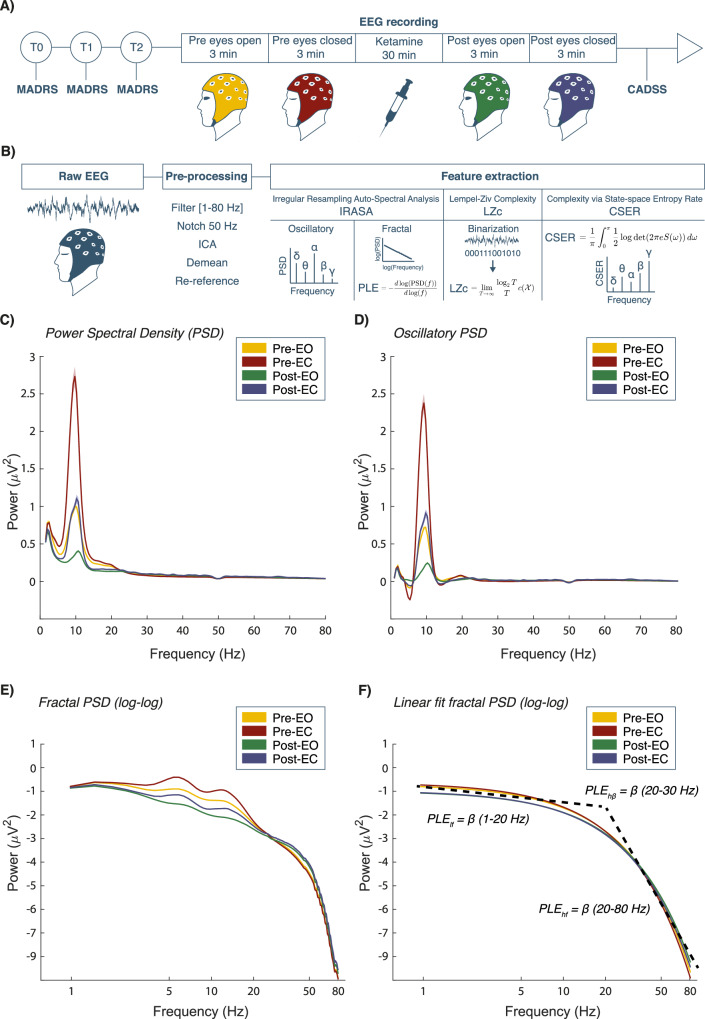
Study design and power spectra. **A** The illustration shows the timeline and desing of the experiment. **B** The flowchart of the EEG analysis steps (see Materials and Methods). **C** The frequency/power plot of the PSD. **D** The isolated oscillatory component of the power spectra obtained with the IRASA method. **E** The log-log plot of the fractal (scale-free) component of the power spectra. **F** The log-log plot of the linear fit of the fractal component of the power spectra. The dashed lines show the interpolated regression lines used to estimate the PLE for the low (1–20 Hz), high frequencies (20–80 Hz), and high β PLE (20–30 Hz). MADRS Montgomery-Åsberg Depression Rating Scale, CADSS Clinician-Administered Dissociative States Scale, Pre-EO pre-ketamine eyes open, Pre-EC pre-ketamine eyes closed, Post-EO post-ketamine eyes open, Post-EC post-ketamine eyes closed. For complexity formulae description refer to [52].

The treatment involved repeated continuous infusions of subanesthetic doses of racemic ketamine (30 min, i.v.) twice a week for a month, with dosage adjusted based on the patient’s needs. The treatment started at dosages below 0.5 mg/kg and was titrated based on individual tolerability and clinical response to a maximum of 1 mg/kg. Depressive symptomatology was monitored by the clinician at baseline (T0) and bi-weekly through the administration of the MADRS until the end of the treatment. Patients who showed a 50% reduction of MADRS after the first week of treatment (T1) were classified as early responders (ER), while the other portion of patients was classified as late responders (LR). For each patient, the EEG recording was performed once, during a ketamine administration with a dosage between 0.5 and 1 mg/kg (T2), known to reliably produce acute subjective effects [47]. The session was scheduled at the next available time after the patient exhibited a clinically significant response to treatment. This scheduling, combined with a dosage sufficient to elicit dissociative effects, was intended to explore potential endophenotypic variability in therapeutic response to ketamine and to investigate the relationship between EEG alterations and dissociative experiences. Importantly, the study was designed to minimize invasiveness and patient distress, given the complexity of this real-world population. At T2 (before the EEG recording), all patients had responded to the treatment (MADRS at T2 < 19). To quantify the subjective experience induced by ketamine on the day of the EEG, the CADSS was administered immediately at the end of the infusion [11]. This scale was chosen as being the most commonly used in previous literature and was adapted to the Italian language and scored into the factors of depersonalization, derealization, and amnesia [11, 48]. A graphical summary of the study design is shown in Fig. 1A.

### EEG recording

The EEG recording consisted of 6 min of baseline resting state before the start of ketamine infusion, composed of 3 min of eyes open (EO) and 3 min of eyes closed (EC) conditions. Immediately at the end of the infusion, EEG recording continued for an additional 6 min, again subdivided into 3 min of EO and 3 min of EC. The EEG recording was performed with a Wireless, 32-channel Starstim device (Neuroelectrics®). The montage included 32 Ag^+^/ Ag^+^Cl^−^ passive electrodes (10–20 international EEG system). The acquisition sampling rate was 500 Hz. The reference electrode was placed on the left mastoid. Data were acquired with the Neuroelectrics®software. More information on the EEG apparatus and setting of the recording are provided in the Supplementary Data.

### EEG pre-processing

The continuous EEG signal was pre-processed offline, retaining the sampling resolution of 500 Hz. The pre-processing steps followed a standard procedure. First, the data were baseline corrected, band-passed between 1 and 80 Hz, and notch filtered at 50 Hz (Butterworth filter). Then, a semi-automatic artifact removal approach was applied. Independent Component Analysis (ICA) was applied to remove EEG components related to muscle activity, blinks, ocular movement, and cardiac activity. The ICA was performed using the “runica” algorithm. A comparable amount of channels (Pre-ketamine: M = 2, SD = 2; Post-ketamine: M = 3, SD = 2) and ICA components (Pre-ketamine: M = 6, SD = 2; Post-ketamine: M = 8, SD = 3) was removed before and after ketamine. Noisy channels were then interpolated using the weighted average of neighboring electrodes. Lastly, the data were re-referenced to the average of all electrodes. The EO and EC conditions had similar data length after pre-processing: Pre-EO (M = 182 s, SD = 7), Pre-EC (M = 177 s, SD = 7), Post-EO (M = 184 s, SD = 11), and Post-EC (M = 175 s, SD = 13). All pre-processing steps and analysis were implemented in Matlab software using the open-source toolbox FieldTrip [49].

### EEG analysis

The pre-processed EEG data were subdivided into non-overlapping epochs of 2 s. For the power spectral density (PSD) analysis, Fast-Fourier transformation was applied using single Hanning taper for low frequencies (1–30 Hz) and multiple tapers for high frequencies (30–80 Hz). To determine the separate contribution of oscillatory and fractal components to the original spectral power, the signal was decomposed using the Irregularly Resampled Auto Spectral Analysis (IRASA) [50]. Original, fractal, and oscillatory spectral power density were divided into the following canonical frequency bands for statistical analysis: δ (1–4 Hz), θ (4–8 Hz), α (8–13 Hz), low β (13–20 Hz), high β (20–30 Hz), low γ (30–45 Hz), and high γ (55–80 Hz). To estimate the PLE of the power spectrum, the fractal component 1/f^PLE^ was transformed to log-log coordinates, and the slope of the distribution was computed using linear regression (Fig. 1D, E). Visual inspection of 1/f distribution revealed a “knee” frequency at 20 Hz (Fig. 1E). Thus, data were separated into two spectral regions, a high-frequency region (PLE_hf_, 20–80 Hz) and a low-frequency region (PLE_lf_, 1–20 Hz) (see ref. [37]). For the quantification of LZc, the data were first binarized by comparing each data point for epoch and channel to the mean value of that channel and epoch. Then, the LZc-76 algorithm was applied to compute the number of distinct “patterns” (or substrings) in each binarized epoch and channel computed as:

1 $${{{\rm{h}}}}\left({{{\rm{X}}}}\right)={\lim}_{{{{\rm{T}}}}\to {{\infty }}}\frac{{\log }_{2}{{{\rm{T}}}}}{{{{\rm{T}}}}}c({{{\rm{X}}}})$$

Where h(X) is the entropy rate, T is the length of the signal, and c(X) represents the number of distinct patterns in the sequence X [51]. The contribution of each frequency band to overall complexity was quantified using the novel estimator Complexity via State-space Entropy Rate (CSER) computed as:

2 $${{{\rm{h}}}}\left({{{\rm{X}}}}\right)=\frac{1}{{{{\rm{\pi }}}}}{\int }_{0}^{{{{\rm{\pi }}}}}\frac{1}{2}{{\mathrm{log\; det}}}(2{{{\rm{\pi }}}}{{{\rm{eS}}}}({{{\rm{\omega }}}})){{{\rm{d}}}}{{{\rm{\omega }}}}$$

Where h(X) is the entropy rate, S(ω) is the spectral density matrix of frequency ω (frequency range from 0 to π), det(S(ω)) is the determinant of the spectral density matrix, 2 πe is a normalization factor, $\frac{1}{{{{\rm{\pi }}}}}$ and $\frac{1}{2}$ are normalization constants to account for the units of frequency and the real-valued nature of the signal (for detailed description see ref. [52]). The open-source EntRate package was used for the LZc and CSER analysis [52]. A graphical flowchart illustrating the EEG feature extraction analysis is presented in Fig. 1B.

### Statistical analysis

The analysis of the original, fractal, and oscillatory spectral power density, as well as the PLE and LZc measures of the EEG, involved channel-specific comparisons between the EO and EC conditions before and after ketamine infusion. For each EEG metric, cluster-level permutation tests were computed between conditions with 1000 random permutations (minimum number of electrodes per cluster = 2, α-level = 0.05) [53]. To analyze changes in CSER, an average CSER value across channels was obtained for each frequency and their sum. A linear mixed-effects model was used to calculate the pre- vs post-ketamine difference in CSER. All analyses were performed on the full dataset as well as between the LR and ER sub-groups. For the correlations among EEG metrics and between EEG metrics with dose, CADSS, and MADRS scores at T2, non-parametric Spearman correlation tests (ρ) were performed using only the electrodes belonging to statistically significant clusters computed with the permutation tests. To account for multiple comparisons, the p values were adjusted independently using the Benjamini-Hochberg adjustment (p adj.) [54]. Also, the confidence of the results was estimated by computing the Bayes Factor (BF). The complete test statistics of each result is reported in the Supplementary data.

## Results

### Demographics, drugs, and psychometric measures

All patients reported a clinically meaningful experience of dissociation during the EEG-recorded ketamine administration, defined by a CADSS total score above 4 [48]. No significant correlations between dose and CADSS total or CADSS subdimensions were observed. Also, there was no correlation between MADRS scores before ketamine administration and CADSS scores after ketamine infusion (Supplementary data).

### EEG metrics

#### Spectral power density (PSD)

**Fig. 2:**
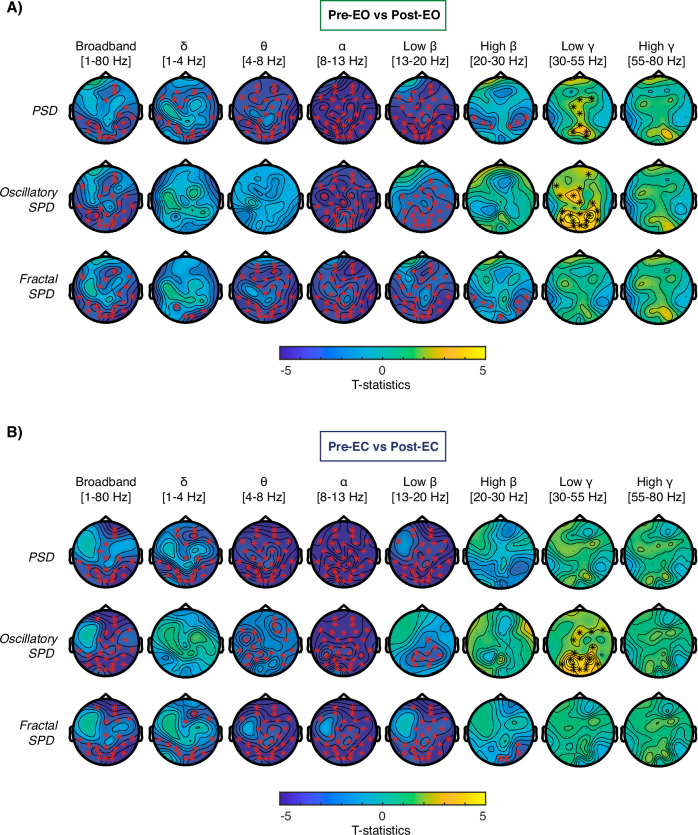
The rhythmic features of the EEG signal. **A** Topographical plots displaying T-statistics for the EO contrast comparing the original, oscillatory, and fractal components of the spectral power density, divided by frequency, between the pre- and post-ketamine conditions. **B** Topographical plots displaying T-statistics for the EC contrast comparing the original, oscillatory, and fractal components of the spectral power density, divided by frequency, between the pre- and post-ketamine conditions. The color bar displays the cluster-based permutation t values. Electrodes belonging to significant clusters are marked in red, if there was a decrease from pre- to post-ketamine, and in black if there was an increase from pre- to post-ketamine. Clusters were considered statistically significant with an α value = 0.025.

Ketamine produced a significant reduction in broadband PSD (Fig. 1C) in both the EO (*p* = 0.002, Fig. 2A) and EC contrasts (*p* < 0.001, Fig. 2B). In particular, the frequency-specific analysis showed a reduction in the δ (EO, *p* < 0.001; EC, *p* = 0.002), θ (EO, *p* < 0.001; EC, *p* < 0.001), α (EO, *p* < 0.001; EC, *p* < 0.001), low β (EO, *p* < 0.001; EC, *p* < 0.001) in both contrasts, and high β in the EO (EO, *p* = 0.016) but not the EC contrast (Fig. 2).

#### Oscillatory component of the power spectra

The analysis of the oscillatory component of the PSD (Fig. 1D) showed a significant broadband reduction following ketamine administration in the EO (*p* < 0.001; Fig. 2A) and EC contrasts (*p* < 0.001; Fig. 2B). Frequency-specific oscillatory power showed significant reductions only for the α (EO, *p* < 0.001; EC, *p* < 0.001) and low β (EO, *p* < 0.001; EC, *p* = 0.014) frequencies, while an increase was observed for the low γ (EO, *p* < 0.001; EC, *p* < 0.001) oscillatory power. There was also a reduction of θ for the EC (*p* = 0.005; Fig. 2B) contrast only.

#### Power-law exponent (PLE)

**Fig. 3:**
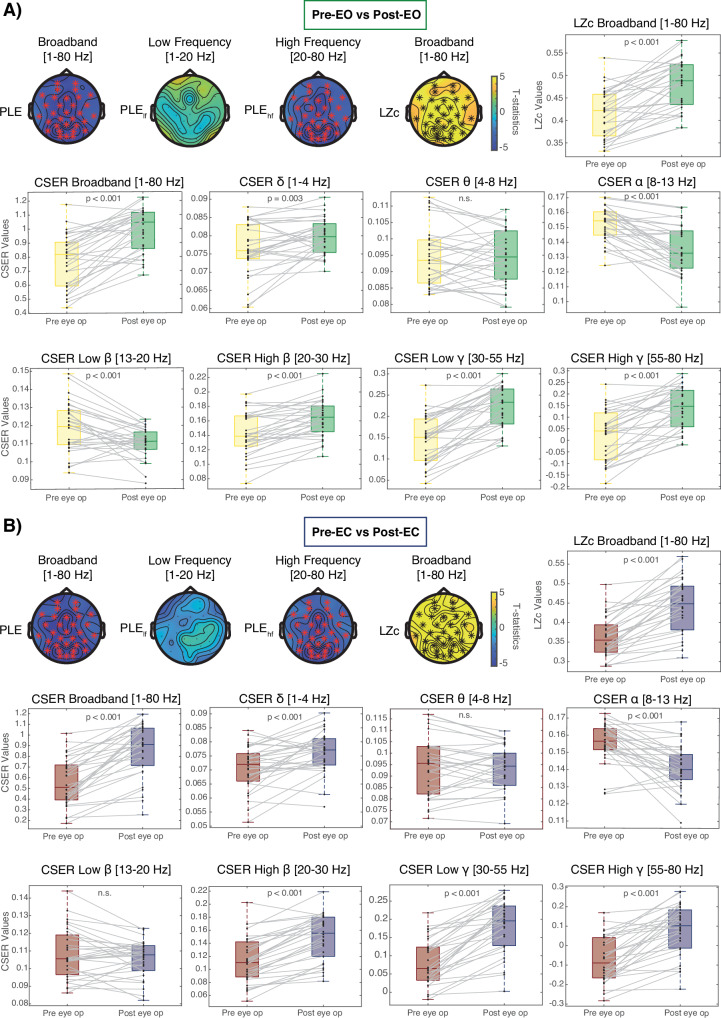
The arrhythmic features of the EEG signal. **A** The difference in arrhythmic features of the EEG signal in the EO condition. **B** The difference in arrhythmic features of the EEG signal in the eyes closed condition. Those include topographical plots displaying the T-statistics of the difference in the PLE_lf_, the PLE_hf_, the normalized LZc, the channel average of the LZc as well as the broadband and frequency-decomposed CSER between pre- to post-ketamine conditions. The color bar displays the cluster-based permutation t values. Electrodes belonging to significant clusters are marked in red, if there was a decrease from pre- to post-ketamine, and in black if there was an increase from pre- to post-ketamine (α = 0.025).

A statistically significant reduction of the broadband PLE was observed following ketamine exposure for both the EO (*p* < 0.001; Fig. 3A) and EC (*p* < 0.001; Fig. 3B) contrasts, signifying a reduction in the steepness of the 1/f fractal distribution. In particular, the difference was specific to frequencies above 20 Hz (PLE_hf_: EO, *p* < 0.001; EC, *p* < 0.001). Further analysis of the PLE_hf_ showed that the change in slope was most significant within the high β frequency band in both contrasts (EO, *p* < 0.001; EC *p* < 0.001). Therefore, the change in PLE within the high β (PLE_hβ_) was used for the correlation with the other EEG and clinical metrics.

#### Signal entropy

Both EO and EC contrasts showed a marked increase in brain signal entropy, as quantified by broadband LZc (EO, *p* < 0.001; EC, *p* < 0.001) and CSER (EO, *p* < 0.001; EC, *p* < 0.001) following ketamine administration (Fig. 3). Frequency-decomposed CSER showed an increase within the δ (EO, *p* = 0.005; EC, *p* < 0.001), high β (EO, *p* < 0.001; EC, *p* < 0.001), low γ (EO, *p* < 0.001; EC, *p* < 0.001), and high γ (EO, *p* < 0.001; EC, *p* < 0.001) bands but a decrease within α (EO, *p* < 0.001; EC, *p* < 0.001) for both contrasts. The CSER within the low β showed a decrease for the EO contrast (*p* < 0.001) but no change for the EC.

### Correlation between EEG metrics

There was a statistically significant negative correlation between relative change in broadband CSER and PLE_hβ_ in the EC contrasts (p adj. = 0.012). Within the α band, the change in CSER and PLE_hβ_ correlated positively (EO, p adj. < 0.001; EC, p adj. = 0.011), as did the change in oscillatory power with PLE_hβ_ in both contrasts (EO, p adj. < 0.001; EC, p adj. = 0.017). In the low γ band, there was a negative correlation between changes in CSER and PLE_hβ_ in the EC contrast (p adj. = 0.019). All data are shown in Table 2.

**Table 2:** Correlation between EEG metrics.

Condition	EEG Metric 1	EEG metric 2	ρ coefficient	*p* value	p adj
Pre-EO vs post-EO	Oscillatory α	PLE_hβ_	0.69	<0.001*	<0.001*
	Oscillatory α	CSER α	0.36	0.05*	0.315
	Oscillatory low β	PLE_hβ_	0.29	0.117	0.328
	Oscillatory low β	CSER low β	−0.01	0.957	0.957
	Oscillatory low γ	PLE_hβ_	0.11	0.545	0.647
	Oscillatory low γ	CSER low γ	0.07	0.714	0.798
	Oscillatory low γ	PLE_hβ_	−0.34	0.071	0.328
	Oscillatory low γ	CSER low γ	−0.19	0.311	0.423
	Oscillatory broadband	PLE_hβ_	0.05	0.774	0.817
	Oscillatory broadband	CSER tot	0.21	0.26	0.414
	PLE_hβ_	CSER tot	−0.30	0.112	0.328
	PLE_hβ_	CSER δ	−0.19	0.301	0.423
	PLE_hβ_	CSER θ	0.23	0.226	0.414
	PLE_hβ_	CSER α	0.68	<0.001*	<0.001*
	PLE_hβ_	CSER β	−0.21	0.262	0.414
	PLE_hβ_	CSER low β	0.16	0.402	0.509
	PLE_hβ_	CSER high β	−0.29	0.121	0.328
	PLE_hβ_	CSER low γ	−0.24	0.202	0.414
	PLE_hβ_	CSER high γ	−0.22	0.249	0.414
Pre-EC vs Post-EC	Oscillatory θ	CSER high β	0.20	0.279	0.456
	Oscillatory θ	CSER θ	−0.13	0.483	0.655
	Oscillatory α	PLE_hβ_	0.52	0.004*	0.017*
	Oscillatory α	CSER α	0.67	<0.001*	0.001*
	Oscillatory low β	PLE_hβ_	−0.20	0.288	0.456
	Oscillatory low β	CSER low β	0.09	0.652	0.774
	Oscillatory low γ	PLE_hβ_	−0.30	0.112	0.236
	Oscillatory low γ	CSER low γ	−0.05	0.805	0.805
	Oscillatory broadband	PLE_hβ_	−0.43	0.018*	0.056
	Oscillatory broadband	CSER tot	0.18	0.334	0.488
	PLE_hβ_	CSER tot	−0.55	0.002*	0.012*
	PLE_hβ_	CSER δ	−0.10	0.587	0.743
	PLE_hβ_	CSER θ	0.06	0.739	0.805
	PLE_hβ_	CSER α	0.57	0.001*	0.011*
	PLE_hβ_	CSER low β	−0.06	0.769	0.805
	PLE_hβ_	CSER high β	−0.27	0.155	0.295
	PLE_hβ_	CSER low γ	−0.50	0.005*	0.019*
	PLE_hβ_	CSER high γ	0.33	0.076	0.206

The results of pairwise Spearman correlations between relative changes in EEG metrics after ketamine. The *p* values were adjusted for multiple comparisons using the using the Benjamini–Hochberg adjustment (p adj.).

*Statistically significant results at α-level = 0.05.

### Correlation between EEG metrics and ketamine dose

There was a negative correlation between the dose of ketamine (0.5–1 mg/kg) delivered during the EEG and relative changes in α (ρ = −0.41, *p* = 0.024, p adj. = 0.192, BF = 5.57) and low β (ρ = −0.36, *p* = 0.049, p adj. = 0.205, BF = 4.43) PSD in the EC contrast. There was a statistically significant negative correlation between dosage and changes in θ (ρ = −0.40, *p* = 0.027, p adj. = 0.192, BF = 0.67) oscillatory activity in the EC contrast. Relative changes of CSER within the α frequency correlated with dose in both contrasts (EO, ρ = −0.49, *p* = 0.006, p adj. = 0.113, BF = 33.31; EC, ρ = −0.41, *p* = 0.024, p adj. = 0.192, BF = 7.50). A similar correlation was found with low β CSER (ρ = −0.39, *p* = 0.031, p adj. = 0.292, BF = 5.32) in the EO contrast only. However, none of the observed correlations between EEG metrics and ketamine dosage survived correction for multiple comparisons.

### Correlation between EEG and psychological metrics

With the exclusion of the outlier on the CADSS, the derealization scores correlated positively with the relative change in δ power in the EC contrast (EC, ρ = 0.48 *p* = 0.009, p adj. = 0.191, BF = 10.29). Scores on CADSS depersonalization correlated negatively with changes in oscillatory low β activity in the EO contrast (ρ = − 0.39 *p* = 0.039, p adj. = 0.776, BF = 2.16). However, both correlations did not survive correction for multiple comparisons. No significant correlations were found between MADRS scores at T2 and changes in EEG metrics in any of the analyzed contrasts (Supplementary data).

### Early vs late responders

**Fig. 4:**
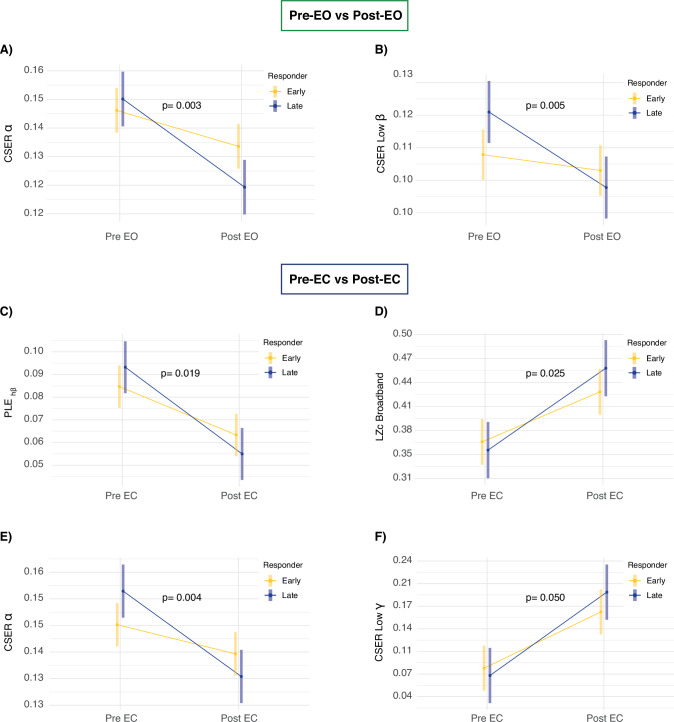
The difference in EEG metrics of ketamine action between early and late responders. **A** The difference in CSER decrease within the α band in the EO condition. **B** The difference in CSER decrease within the low β band in the EO condition. **C** The difference in PLE_β_ reduction in the EC condition. **D** The difference in normalized Lempel–Ziv complexity (LZc) increase in the EC condition. **E** The difference in CSER decrease within the α band in the EC condition. **F** The difference in CSER increase within the low γ band in the EC condition. Significance levels for the interaction effects were considered with α = 0.05. All p values are corrected for ketamine dose.

After correcting for ketamine dose, the LR group showed a steeper decrease in PLE_hβ_ as compared to the ER group in the EC contrast (Β = 0.02, *p* = 0.019; Fig. 4C), with a similar, but not significant, trend in the EO contrast (Β = 0.01, *p* = 0.077). The LR group showed a steeper increase in LZc as compared to the ER group following ketamine exposure in the EC contrast (Β = −0.04, *p* = 0.025; Fig. 4D). Further, the LR group had a steeper decrease in CSER within the α band in both contrasts (EO, Β = 0.01, *p* = 0.003; Fig. 4A; EC, Β = 0.02, *p* = 0.004; Fig. 4E) and of low β CSER (Β = 0.01, *p* = 0.005; Fig. 4B) in the EO contrast, as compared to the ER. The LR group had also a steeper increase in the low γ CSER (Β = −0.04, *p* = 0.050; Fig. 4F) as compared to the ER group in the EC contrast. No statistically significant interactions were observed between changes in PSD and oscillatory activity between ER and LR in any of the analyzed contrasts.

## Discussion

We investigated the neurophysiological changes induced by a subanesthetic dose of ketamine in a BD patient undergoing treatment for depression in a real-world, hospital, setting. We observed an intricate pattern of brain-wide EEG changes induced by ketamine across various rhythmic and arrhythmic signal features.

Analysis of the oscillatory component of the EEG spectra revealed that ketamine predominantly diminished θ, α, and low β activity while increasing low γ. Our results align with previous results in both healthy and depressed individuals [18–23, 30–32]. According to the “disinhibition” model, ketamine preferentially antagonizes N-methyl-D-aspartate (NMDA) receptors expressed on γ-Aminobutyric acid (GABA) inhibitory interneurons, leading to the interruption of local cortical circuit firing. This produces a desynchronization of slow rhythmic activity and elevation of high-frequency activity, as substantiated by in-vitro, in-vivo, and in-silico evidence [55–58]. Consequently, alterations in low and fast EEG rhythms are posited to result from a shift in the cortical excitatory/inhibitory balance toward higher excitability, serving as a crucial index of perceptual, cognitive, and emotional functioning within the brain and a potential biomarker of ketamine antidepressant effect [59, 60].

Ketamine also modulated fractal (scale-free) arrhythmic activity, particularly in the low-to-high frequency transition point. We observed a “flattening” of the slope of the power spectra following ketamine administration, evident in both eyes open and closed conditions. This is consistent with the only available study having quantified the PLE with ketamine in healthy subjects, a finding that we have successfully replicated in a clinical population [37]. The specificity of the effect above the “knee” frequency of 20 Hz in our data is in striking accordance with the modeling work of Gao et al. [61]. They observed that the slope of the 1/f distribution of the fractal component of the spectrum, simulated by an excitatory and an inhibitory neural population, correlates with the excitatory/inhibitory ratio for frequencies above 20 Hz [61]. This raises the intriguing possibility that the alterations in the slope of the scale-free component of the power spectra are influenced by ketamine’s glutamatergic action, potentially serving as a shared factor contributing to the observed rhythmic and arrhythmic changes. While further research is necessary to substantiate this conclusion, our results suggest a potential role for the scale-free properties of EEG as biomarkers for the antidepressant effects of ketamine.

The analysis of the dynamic non-linear properties of the EEG signal revealed that ketamine-induced a widespread increase in the entropy of brain activity, replicating previous observations obtained in healthy and depressed individuals [39, 42, 62, 63]. Additionally, a novel measure of informational complexity was applied to achieve spectral decomposition of signal entropy [52]. The analysis revealed that ketamine increased entropy primarily in the high frequencies, including high β and γ. In contrast, signal entropy tended to decrease in lower frequencies, especially in α and low β. These results are akin to the study by Mediano et al. in healthy individuals following acute administration of classic psychedelics [52]. Our study represents the first application of the novel estimator CSER to investigate the effects of ketamine on spectrally decomposed neural entropy. While little is known about their neurophysiological significance, complexity measures of neural activity have been found to be altered in several neuropsychiatric conditions, with reductions observed in depressive and bipolar disorders, albeit not consistently across studies [64]. Our findings align with the idea that heightened complexity may contribute to the mechanism of action of ketamine [65].

The reduction in low-frequency oscillatory power and the flattening of the slope of the fractal component of the spectra induced by ketamine were both associated with the increase in signal entropy. Interestingly, a correlation between reduction in α oscillatory power and PLE was observed, supporting the computational model proposed by Muthukumaraswamy and Liley [37]. Notably, the reduction in brain entropy within α and the increase in γ frequencies were also associated with changes in PLE. This tripartite relationship between oscillatory, fractal, and entropic properties of the EEG potentially introduces a novel effect of ketamine manifested by the interaction between rhythmic and arrhythmic dynamics of the brain signal.

Taken together, the observed EEG effects of ketamine indicate a global shift in excitatory/inhibitory balance induced by the glutamatergic action of ketamine, potentially underpinning the altered state of consciousness produced by subanesthetic doses of ketamine. However, our analysis of the relationship between the modulation of EEG and reported experiences of dissociation induced by ketamine yielded mixed results. We observed a negative correlation between oscillatory activity in the low β with experiences of depersonalization and an unexpected positive correlation between changes in δ power and derealization, but both results were not robust against correction for multiple comparisons. Additionally, no significant associations were found between the CADSS and arrhythmic components of the EEG. Limitations of the study, such as the small sample size, the use of a non-validated translation of the scale, or the limited adequateness of the scale to capture the full breadth of ketamine’s acute subjective effect, might potentially explain the lack of consistent results. Comparisons of the CADSS with qualitative reports of the subjective experience induced by ketamine have indicated that the scale fails to capture important themes of the experience, and low scores on the CADSS were often associated with reports of clinically significant drug effects [11]. These limitations may stem from the fact that the CADSS was originally developed to capture symptoms of dissociation in conditions such as dissociative disorders and trauma [48], possibly making it unsuitable for capturing the specific alteration of consciousness induced by ketamine. While the CADSS was selected for its practicality and widespread use in clinical research on ketamine, the inclusion of other scales commonly used in psychedelic research, like the ASCQ, could have added greater nuance to the study. Also, future research into ketamine action should consider adopting the neurophenomenological approach to altered states of consciousness proposed by Timmerman et al., possibly revealing fine-graded and specific properties of the drug-induced experience [66]. However, the application of thorough, yet time-consuming, methods for investigating subjective experience remains challenging in real-world, hospitalized settings.

A key finding of this study was the distinct neurophysiological responses to ketamine between early and late responders. Intriguingly, LR to ketamine treatment exhibited a greater acute modulation of EEG features compared to ER. These effects were not attributable to differences in ketamine dosage, baseline EEG markers (Supplementary Data), or depression severity at the time of EEG recording. Notably, the clinically relevant effects observed in this study were specifically linked to the arrhythmic properties of the EEG signal, highlighting the functional relevance of these metrics. Given that LR received more ketamine exposures at the time of EEG measurement due to the delayed clinical response, one might expect their neurophysiological response upon drug exposure to be diminished compared to ER. However, the counterintuitive finding of heightened EEG modulation in this group suggests that LR may undergo more profound neural changes compared to ER. This raises the possibility that individual differences in the underlying pathological endophenotype could account for varying sensitivities to ketamine’s effects, possibly rooted in latent neural network dynamics [67]. There is some evidence suggesting that depressive endophenotypes characterized by more severe neural dysregulation experience more pronounced changes following ketamine administration [68, 69]. For instance, in a study measuring synaptic density post-ketamine treatment it was shown that patients with lower baseline synaptic density exhibit greater increases, which correlated with reductions in depression severity and increases in dissociative symptoms [69]. At the same time, it is plausible that the repeated exposure to ketamine in LR may promote more substantial neuroadaptive processes, such as enhanced synaptic plasticity and widespread network reorganization [70]. While not directly comparable, a recent finding suggest that increases in synaptic density following SSRI treatment emerge only after prolonged drug exposure [71]. Thus, these neuroadaptive changes may require more time to manifest in LR as compared to ER and may not be immediately reflected in clinical scales. If so, the arrhythmic features of the EEG signal could serve as a neuropharmacological index, reflecting ongoing neuroadaptive brain processes. However, given the exploratory nature of the finding, such interpretations remain speculative, and the result require replication in larger samples, with repeated and multimodal assessments in controlled settings to draw more definitive conclusions. Furthermore, research into the non-linear aspects of brain activity is still in its early stages, and their biological significance remains to be fully understood.

It is important to recognize the naturalistic design of the study, which presents both strengths and weaknesses. On one hand, the study provides new insights into ketamine’s neurophysiological effects in complex BD patients within a real-world clinical environment. However, factors such as variations in prior exposures to ketamine before EEG recordings, as well as the diverse and concurrent poly-pharmacological treatments received by patients, limit the interpretability and generalizability of our findings. Nonetheless, the replication of numerous previous findings in controlled settings highlights the robustness of our approach.

In summary, the study offers comprehensive insights into the acute neurophysiological effects of ketamine in patients with BD undergoing treatment for depression. The observed alterations across various rhythmic and arrhythmic components of brain signals underscore the potential usefulness of EEG as a valuable tool for evaluating and monitoring the neurobiological effects of ketamine in real-world clinical settings. Additionally, the distinct responses observed between early and late responders emphasize the clinical significance of endophenotypic differences in response to psychoactive medications, reinforcing the need for further research in this area. If replicated, our findings could contribute to advancing clinical protocols for ketamine by allowing for the stratification of patients based on their neurophysiological response to the drug. This would enable more personalized treatment approaches, where ketamine therapy could be tailored to individual patient profiles, optimizing dosage and treatment duration.

## Supplementary information

Supplementary Data

## Data Availability

Data and codes are available upon direct request to the authors. Address correspondence to Claudio Agnorelli at claudio.agnorelli@student.unisi.it or claudioagnorelli96@gmail.com.
