# Supplementary material for: Neurophysiological correlates of ketamine-induced dissociative state in bipolar disorder: insights from real-world clinical settings
Source: Mol Psychiatry. 2025 Jan 14;30(7):2848–59. doi: 10.1038/s41380-025-02889-2 (PMC12185349; doi:10.1038/s41380-025-02889-2)
Supplement: Supplementary file 1 — Supplementary Data [file 41380_2025_2889_MOESM1_ESM.docx]

Supplementary methods

EEG recording

EEG recordings were performed using a 32-channel EEG system (Starstim; Neuroelectrics, Barcelona, Spain). The device was connected by cable to the laptop. Hybrid electrodes (NG Pistim) were used, consisting of an upper part containing the sintered Ag/AgCl core with a diameter of 12 mm, screwed onto a lower base such that a circular area of about 3.14 cm2 was covered. The electrodes were placed on a 32-channel neoprene EEG headset with holes prepared according to the International 10-20 EEG System. The scalp portion below the electrode was prepared by inserting 15 ml of sterile sodium chloride solution (0.9%) and Gel (Signa, Parker Laboratories, Inc.) to avoid uncomfortable sensations on the skin and reduce impedances. Electrode impedance was checked before starting each EEG recording and was kept below 10 kΩ. Once the headset was fitted, subjects were asked to lie on a bed with an inclined backrest according to their comfort. The NIBS protocol involves a pre-infusion resting state EEG recording characterised by a duration of six minutes of which three minutes with eyes open and three minutes with eyes closed. The anesthetist was then asked to set up the thirty minute infusion and at the end of the infusion another six minute resting EEG recording was made (three minutes eyes open, three minutes eyes closed). During the infusion, subjects were asked to remain silent with their eyes open for the entire duration. Music listening was not allowed during the whole EEG recording. For the convenience of the subjects, the Starstim device was placed on a table prepared for this purpose. The only active electrical device in the room was the ketamine infusion device. The laptop with which the EEG recording was made was battery-powered and there were no other electrical devices in the room. For the duration of all recordings only the subject and the experimenter were present in the room. The room was approximately 10sqm, indirectly lit by sunlight and artificial lights were kept off for all subjects. The time of administration of the protocol was restricted to the 3:00 pm to 6:00 pm time slot. Only five subjects registered in the morning between 9:00 am and 11:00 am.

EEG analysis

All analysis were performed on the clean EEG data recording of the 3 minutes eye open and 3 minutes eyes closed conditions before and immediately following ketamine infusion. Prior to all analysis, the continuous pre-processed EEG data were subdivided into non-overlapping epochs of 2 seconds.

For the power spectral density (PSDPSD) analysis, data were Fast-Fourier transformed using a conventional single taper (e.g., Hanning) for frequencies between 1 and 30 Hz and using the multiple tapers based on discrete prolate spheroidal sequences for frequencies between 30 and 80 Hz.

To determine the separate contribution of oscillatory and fractal components to the original spectral power, the signal was decomposed using the Irregularly Resampled Auto Spectral Analysis (IRASA) algorithm, as described by Wen and Liu (1). The technique virtually compresses and expands the time-domain data with a set of non-integer resampling factors prior to FFT-based spectral decomposition. As a result, oscillatory components in the power-spectrum are redistributed while the fractal 1/f contribution is left intact. Taking the median of the resulting auto-spectral distributions extracts the power-spectral fractal component, and the subsequent removal of the fractal component from the original power-spectrum offers a power-spectral estimate of oscillatory content alone.

to estimate the PLE of the power spectrum, the fractal component 1/f^Β^ was transformed to log-log coordinates. Taking the log brings the Β down from the exponent, turning the relation to a linear one: log(1/f Β) = Β log(1/f). Then, a linear regression was performed to extract the β coefficient. Finally, taking the negative of the Β coefficient turns f^Β^ to 1/f^Β^ (i.e., PLE). To avoid biasing regression estimates towards the higher frequencies, where more sampling points exist in logarithmic space, frequency estimates are resampled to be evenly spaced in logarithmic coordinates prior to computation of the regression. Also, visual inspection of the log-log distribution of the fractal power showed a "knee" frequency at 20 Hz, were the slope (i.e., Β coefficient or PLE) of the spectrum showed a significant change. Thus, data were separated into frequency bands "by eye" into two spectral regions, a high-frequency region (20-80 Hz) for which the parameter PLE_hf_ was defined and a lower frequency range (1-20 Hz) for which PLE_lf_ was defined, following a similar approach to the one of Muthukumaraswamy and Liley (2). Based on the analysis reported in the main results, a PLE_β_ was defined for the high β frequency.

For the quantification of LZc, the pre-processed data were first binarized by comparing each data point for epoch and channel to the mean value of that channel and epoch, with values above the mean transformed into 1s and values below into 0s. Then, the LZc 76 algorithm was applied to compute the number of distinct "patterns" (or substrings) in each binarized epoch and channel, and then normalized the resulting number for data length by a factor N/log2(N), where N is the data length in samples. Then, the normalized values were averaged over epochs to obtain a measure of brain entropy estimate (here referred as LZc) . Regular signals are characterized by a small number of patterns and hence have low LZc, while irregular signals contain many different patterns and hence have a high LZc. The analysis followed the original method described by Lempel A, Ziv J (3), applied to EEG data.

For the quantification of complexity contribution of each frequency band, the novel estimator called Complexity via State-space Entropy Rate (CSER) was computed. The CSER is a spectrally and temporally resolved estimation of neural signal diversity, recently introduced by Mediano et al (4). Compared to LZc, CSER does not require the signal to be discretized, allowing it to fully exploit continuous signals and avoiding potential artefacts introduced by the discretization procedure. Also, CSER has the unique advantage of allowing for complexity analysis within spectral frequency bands. First, the pre-processed data were down sampled to 160 Hz. Then data were normalized via z-scoring, by subtracting the mean and then dividing by the standard deviation in each channel. The CSER was applied to the z-scored data to obtain a CSER measure for each frequency band, which adds up to a total CSER. The frequency-specific CSER was computed on 2 Hz windows of the broadband and then averaged within the frequency bands of interest. For comparison, we replicated the LZc analysis with the same down-sampled to 160 Hz and normalized data, showing comparable results to the LZc computed on the 500 Hz resolution data (Supplementary Table 6). All analysis were performed with Matlab software.

Statistical analysis

All EEG metrics were computed as temporally averaged within each condition, 3 minutes-long eyes open (EO) and eyes closed (EC) pre- and post-ketamine. The analysis of the original, fractal, and oscillatory spectral power density (divided in the main frequencies of interest), as well as the PLE and LZc measures of the EEG, involved group-level channel-specific comparisons between the EC and EO conditions. For each EEG metric and channel, a cluster-level permutation tests was computed between conditions, an approach shown to be the most efficient in addressing the multiple comparisons problem (5). For every channel, the experimental conditions are compared by means of a t-value. All samples are selected whose t-value is larger than a cluster α value of 0.05. Then, selected samples are clustered in connected sets on the basis of temporal, spatial and spectral adjacency. Cluster-level statistics are calculated by taking the sum of the t-values within every cluster and the maximum of the cluster-level statistics is taken. The significance probability is calculated by means of the so-called Monte Carlo method. The method collect the trials of the different experimental conditions in a single set and randomly draw as many trials from this combined data set as there were trials in condition 1 and place them into subset 1. The remaining trials are placed in subset 2. On this randomized set, the test statistic is computed as described above (i.e., the maximum of the cluster-level summed t-values). These steps were repeated for 1000 random permutations to obtain a distribution of test statistics, from which the proportion of random partitions that resulted in a larger test statistic than the observed one is computed. If the probability value (i.e., p value) is smaller than the critical α-level of 0.025 (one tail for positive and one for negative clusters; total 0.05), then the data in the two experimental conditions were considered significantly different.

To analyse changes in CSER, an average CSER value across channels was obtained for each frequency and for the broadband data. A linear mixed-effect model was used to calculate the pre- vs post-ketamine difference in CSER, using a random slope for each subject to account for the within-subject design. This analysis was performed using the open-source programming language R.

For correlations between EEG metrics and between EEG metrics with dose, scores on the CADSS, and MADRS scores at T2, the electrodes belonging to statistically significant clusters computed with the permutation tests were extracted for each comparison and metric. Then, the difference between post- to pre-ketamine values was computed for those electrodes only. Both the raw and relative (corrected for the baseline value) differences were computed. For each contrast, non-parametric Spearman correlation tests (ρ) were performed between EEG metrics, total, depersonalization, derealization, and amnesia CADSS scores, MADRS scores at T2 (EEG day, before ketamine administration), and ketamine dosage. For the correlation with the CADSS scores only the analysis was performed after the exclusion of 1 patient who reported an abnormally high CADSS score compared to the others. The outlier was identified via analysis on the quantile distribution using the Tukey’s rule (6). Outliers were defined as data points laying 1.5 times the Interquartile range above the third quartile (75% of the distribution) or below the first quartile (25% of the distribution). To account for FDR inflation due to multiple comparisons, the p values resulting from spearman tests were adjusted independently using the Benjamini-Hochberg adjustment (7). Results of the false discovery rate correction are reported as "p adj. " in the main text.

For the analysis of early versus late responders, patients were stratified based on the difference in MADRS scores between baseline and 1 week of treatment. Patients who showed a reduction of MADRS scores of more than 50 % between the 2 time points where considered early responders (ER), while the others were classified as late responders (LR). The significance analysis for the comparison of baseline EEG metrics between groups was performed with cluster-based permutation statistics with a between-subject design. For the comparison of EEG changes induced by ketamine between groups, cluster-based permutation statistics with a mixed within-between subject design was employed. First, condition differences were computed for each EEG metric and contrasts (i.e., post-EO vs pre-EO; post-EC vs pre-EC). Then the difference between condition was compared between groups (i.e., ER vs LR). For CSER and for the inclusion of dosage as a covariate, the analysis was implemented with a linear mixed-effects model, using the channel-averaged EEG metrics as dependent variable, group and condition as independent variables, plus their interaction, dose as covariate, and a random effect for each subject.

Supplementary Results

Correlations between psychological metrics

|  | | **MADRS – EEG (T2)** | **CADSS Broadband** | **CADSS amnesia** | **CADSS derealization** | **CADSS depersonalization** | **Ketamine Dose (mg/kg)** |
| --- | --- | --- | --- | --- | --- | --- | --- |
| **MADRS – EEG (T2)** | ρ coefficient | - | 0.12 | 0.25 | 0.16 | -0.04 | -0.06 |
|  | p value | - | 0.534 | 0.185 | 0.408 | 0.818 | 0.771 |
| **CADSS Broadband** | ρ coefficient | 0.12 | - | 0.56 | 0.89 | 0.82 | -0.14 |
|  | p value | 0.534 | - | 0.001* | < 0.001* | < 0.001* | 0.461 |
| **CADSS amnesia** | ρ coefficient | 0.25 | 0.56 | - | 0.39 | 0.40 | -0.14 |
|  | p value | 0.185 | 0.001* | - | 0.031* | 0.027* | 0.457 |
| **CADSS derealization** | ρ coefficient | 0.16 | 0.89 | 0.39 | - | 0.63 | -0.11 |
|  | p value | 0.408 | < 0.001* | 0.031* | - | < 0.001* | 0.558 |
| **CADSS depersonalization** | ρ coefficient | -0.04 | 0.82 | 0.40 | 0.63 | - | -0.22 |
|  | p value | 0.818 | < 0.001* | 0.027* | < 0.001* | - | 0.242 |
| **Ketamine Dose (mg/kg)** | ρ coefficient | -0.06 | -0.14 | -0.14 | -0.11 | -0.22 | - |
|  | p value | 0.771 | 0.461 | 0.457 | 0.558 | 0.242 | - |

Table S1: Correlations between psychological metrics. *Spearman correlation test (ρ) with α-level = 0.05.

Pre- vs post-ketamine EEG metrics

| **Comparison** | **Frequency** | **Cluster** | **p value** | **Cluster-Stat** | **Std-Dev** | **CI-Range** |
| --- | --- | --- | --- | --- | --- | --- |
| Pre-EC vs Pre-OP | δ | Positive | 0.012* | 32.228 | 0.003 | 0.007 |
| Pre-EC vs Pre-OP | θ | Positive | 0.001* | 158.471 | 0.001 | 0.002 |
| Pre-EC vs Pre-OP | α | Positive | 0.001* | 189.748 | 0.001 | 0.002 |
| Pre-EC vs Pre-OP | β1 | Positive | 0.002* | 87.282 | 0.001 | 0.003 |
| Pre-EC vs Pre-OP | high β | Positive | 0.008* | 26.140 | 0.003 | 0.006 |
| Pre-EC vs Pre-OP | high β | Negative | 0.037 | -11.745 | 0.006 | 0.012 |
| Pre-EC vs Pre-OP | low γ | Negative | 0.024* | -18.467 | 0.005 | 0.009 |
| Pre-EC vs Pre-OP | high γ | Negative | 0.045 | -10.419 | 0.007 | 0.013 |
| Pre-EC vs Pre-OP | broadband | Positive | 0.001* | 103.314 | 0.001 | 0.002 |
| Pre-EC vs Pre-OP | δ | None | 1 | - | - | - |
| Post-EC vs Post-OP | θ | Positive | 0.007* | 78.991 | 0.003 | 0.005 |
| Post-EC vs Post-OP | α | Positive | 0.001* | 121.079 | 0.001 | 0.002 |
| Post-EC vs Post-OP | β1 | Positive | 0.002* | 58.969 | 0.001 | 0.003 |
| Post-EC vs Post-OP | high β | None | 1 | - | - | - |
| Post-EC vs Post-OP | low γ | Negative | 0.020* | -15.572 | 0.004 | 0.009 |
| Post-EC vs Post-OP | high γ | Negative | 0.037 | -7.948 | 0.006 | 0.012 |
| Post-EC vs Post-OP | broadband | Positive | 0.017* | 28.320 | 0.004 | 0.008 |
| Post-EO vs Pre-OP | δ | Negative | 0.001* | -43.333 | 0.001 | 0.002 |
| Post-EO vs Pre-OP | θ | Negative | 0.001* | -108.082 | 0.001 | 0.002 |
| Post-EO vs Pre-OP | α | Negative | 0.001* | -191.301 | 0.001 | 0.002 |
| Post-EO vs Pre-OP | β1 | Negative | 0.001* | -153.625 | 0.001 | 0.002 |
| Post-EO vs Pre-OP | high β | Negative | 0.016* | -15.723 | 0.004 | 0.008 |
| Post-EO vs Pre-OP | low γ | Positive | 0.028 | 8.612 | 0.005 | 0.010 |
| Post-EO vs Pre-OP | high γ | Positive | 0.038 | 7.822 | 0.006 | 0.012 |
| Post-EO vs Pre-OP | broadband | Negative | 0.002* | -53.795 | 0.001 | 0.003 |
| Post-EC vs Pre-EC | δ | Negative | 0.002* | -67.920 | 0.001 | 0.003 |
| Post-EC vs Pre-EC | θ | Negative | 0.001* | -151.569 | 0.001 | 0.002 |
| Post-EC vs Pre-EC | α | Negative | 0.001* | -221.263 | 0.001 | 0.002 |
| Post-EC vs Pre-EC | β1 | Negative | 0.001* | -152.812 | 0.001 | 0.002 |
| Post-EC vs Pre-EC | high β | Negative | 0.041 | -9.875 | 0.006 | 0.012 |
| Post-EC vs Pre-EC | low γ | None | 1 | - | - | - |
| Post-EC vs Pre-EC | high γ | None | 1 | - | - | - |
| Post-EC vs Pre-EC | broadband | Negative | 0.001* | -74.758 | 0.001 | 0.002 |

Table S2: Pre- vs post-ketamine Spectral Power Density (PSD). *Cluster-based permutation test with α-level = 0.025 for each cluster (total α-level = 0.05).

| **Comparison** | **Frequency** | **Cluster** | **p value** | **Cluster-Stat** | **Std-Dev** | **CI-Range** |
| --- | --- | --- | --- | --- | --- | --- |
| Pre-EC vs Pre-EO | δ | None | 1 | - | - | - |
| Pre-EC vs Pre-EO | θ | Positive | 0.001* | 70.945 | 0.001 | 0.002 |
| Pre-EC vs Pre-EO | α | Positive | 0.001* | 141.754 | 0.001 | 0.002 |
| Pre-EC vs Pre-EO | β1 | Negative | 0.001* | -66.332 | 0.001 | 0.002 |
| Pre-EC vs Pre-EO | high β | Negative | 0.056 | -7.331 | 0.007 | 0.014 |
| Pre-EC vs Pre-EO | low γ | Negative | 0.013* | -17.187 | 0.004 | 0.007 |
| Pre-EC vs Pre-EO | high γ | Negative | 0.033 | -7.930 | 0.006 | 0.011 |
| Pre-EC vs Pre-EO | broadband | Positive | 0.001* | 141.892 | 0.001 | 0.002 |
| Post-EC vs Post-EO | δ | None | 1 | - | - | - |
| Post-EC vs Post-EO | θ | None | 1 | - | - | - |
| Post-EC vs Post-EO | α | Positive | 0.001* | 82.768 | 0.001 | 0.002 |
| Post-EC vs Post-EO | β1 | Negative | 0.001* | -33.428 | 0.001 | 0.002 |
| Post-EC vs Post-EO | HIGH Β | None | 1 | - | - | - |
| Post-EC vs Post-EO | low γ | Negative | 0.001* | -31.969 | 0.001 | 0.002 |
| Post-EC vs Post-EO | high γ | None | 1 | - | - | - |
| Post-EC vs Post-EO | broadband | Positive | 0.009* | 47.820 | 0.003 | 0.006 |
| Post-EO vs Pre-EO | δ | None | 1 | - | - | - |
| Post-EO vs Pre-EO | θ | None | 1 | - | - | - |
| Post-EO vs Pre-EO | α | Negative | 0.001* | -137.013 | 0.001 | 0.002 |
| Post-EO vs Pre-EO | β1 | Negative | 0.001* | -73.767 | 0.001 | 0.002 |
| Post-EO vs Pre-EO | HIGH Β | None | 1 | - | - | - |
| Post-EO vs Pre-EO | low γ | Positive | 0.001* | 64.727 | 0.001 | 0.002 |
| Post-EO vs Pre-EO | high γ | Positive | 0.036 | 7.020 | 0.006 | 0.012 |
| Post-EO vs Pre-EO | broadband | Negative | 0.001* | -80.655 | 0.001 | 0.002 |
| Post-EC vs Pre-EC | δ | None | 1 | - | - | - |
| Post-EC vs Pre-EC | θ | Negative | 0.007* | -47.718 | 0.003 | 0.005 |
| Post-EC vs Pre-EC | α | Negative | 0.001* | -162.715 | 0.001 | 0.002 |
| Post-EC vs Pre-EC | β1 | Negative | 0.010* | -26.541 | 0.003 | 0.006 |
| Post-EC vs Pre-EC | high β | None | 1 | - | - | - |
| Post-EC vs Pre-EC | low γ | Positive | 0.001* | 53.976 | 0.001 | 0.002 |
| Post-EC vs Pre-EC | high γ | None | 1 | - | - | - |
| Post-EC vs Pre-EC | broadband | Negative | 0.001* | -119.298 | 0.001 | 0.002 |

Table S3: Pre- vs post-ketamine Irregularly Resampled Auto Spectral Analysis (IRASA) oscillatory component. *Cluster-based permutation test with α-level = 0.025 for each cluster (total α-level = 0.05).

| **Comparison** | **Frequency** | **Cluster** | **p value** | **Cluster-Stat** | **Std-Dev** | **CI-Range** |
| --- | --- | --- | --- | --- | --- | --- |
| Pre-EC vs Pre-EO | δ | Positive | 0.003* | 36.288 | 0.002 | 0.003 |
| Pre-EC vs Pre-EO | θ | Positive | 0.001* | 153.770 | 0.001 | 0.002 |
| Pre-EC vs Pre-EO | α | Positive | 0.001* | 166.941 | 0.001 | 0.002 |
| Pre-EC vs Pre-EO | β1 | Positive | 0.001* | 132.879 | 0.001 | 0.002 |
| Pre-EC vs Pre-EO | high β | Positive | 0.004* | 33.331 | 0.002 | 0.004 |
| Pre-EC vs Pre-EO | low γ | Negative | 0.025* | -10.104 | 0.005 | 0.010 |
| Pre-EC vs Pre-EO | high γ | Negative | 0.033 | -7.579 | 0.006 | 0.011 |
| Pre-EC vs Pre-EO | broadband | Positive | 0.001* | 122.056 | 0.001 | 0.002 |
| Post-EC vs Post-EO | θ | Positive | 0.002* | 83.867 | 0.001 | 0.003 |
| Post-EC vs Post-EO | α | Positive | 0.001* | 87.831 | 0.001 | 0.002 |
| Post-EC vs Post-EO | β1 | Positive | 0.002* | 53.771 | 0.001 | 0.003 |
| Post-EC vs Post-EO | high β | None | 1 | - | - | - |
| Post-EC vs Post-EO | low γ | None | 1 | - | - | - |
| Post-EC vs Post-EO | high γ | None | 1 | - | - | - |
| Post-EC vs Post-EO | broadband | Positive | 0.007* | 35.240 | 0.003 | 0.005 |
| Post-EO vs Pre-EO | δ | Negative | 0.017* | -11.897 | 0.004 | 0.008 |
| Post-EO vs Pre-EO | θ | Negative | 0.001* | -105.116 | 0.001 | 0.002 |
| Post-EO vs Pre-EO | α | Negative | 0.001* | -125.300 | 0.001 | 0.002 |
| Post-EO vs Pre-EO | β1 | Negative | 0.001* | -99.452 | 0.001 | 0.002 |
| Post-EO vs Pre-EO | high β | Negative | 0.020* | -17.383 | 0.004 | 0.009 |
| Post-EO vs Pre-EO | low γ | None | 1 | - | - | - |
| Post-EO vs Pre-EO | high γ | Positive | 0.035 | 7.592 | 0.006 | 0.011 |
| Post-EO vs Pre-EO | broadband | Negative | 0.001* | -66.563 | 0.001 | 0.002 |
| Post-EC vs Pre-EC | δ | Negative | 0.002* | -37.812 | 0.001 | 0.003 |
| Post-EC vs Pre-EC | θ | Negative | 0.001* | -135.597 | 0.001 | 0.002 |
| Post-EC vs Pre-EC | α | Negative | 0.001* | -145.816 | 0.001 | 0.002 |
| Post-EC vs Pre-EC | β1 | Negative | 0.001* | -108.317 | 0.001 | 0.002 |
| Post-EC vs Pre-EC | high β | Negative | 0.021* | -9.932 | 0.005 | 0.009 |
| Post-EC vs Pre-EC | low γ | None | 1 | - | - | - |
| Post-EC vs Pre-EC | high γ | None | 1 | - | - | - |
| Post-EC vs Pre-EC | broadband | Negative | 0.001* | -85.419 | 0.001 | 0.002 |

Table S4: Pre- vs post-ketamine Irregularly Resampled Auto Spectral Analysis (IRASA) fractal component. *Cluster-based permutation test with α-level = 0.025 for each cluster (total α-level = 0.05).

| **Comparison** | **Frequency** | **Cluster** | **p value** | **Cluster-Stat** | **Std-Dev** | **CI-Range** |
| --- | --- | --- | --- | --- | --- | --- |
| Pre-EC vs Pre-EO | PLE_Broadband_ | Positive | 0.001* | 119.037 | 0.001 | 0.002 |
| Post-EC vs Post-EO | PLE_Broadband_ | Positive | 0.002* | 82.618 | 0.001 | 0.003 |
| Post-EO vs Pre-EO | PLE_Broadband_ | Negative | 0.001* | -170.148 | 0.001 | 0.002 |
| Post-EC vs Pre-EC | PLE_Broadband_ | Negative | 0.001* | -166.761 | 0.001 | 0.002 |
| Pre-EC vs Pre-EO | PLE_lf_ | Positive | 0.016* | 38.138 | 0.004 | 0.008 |
| Post-EC vs Post-EO | PLE_lf_ | Positive | 1 | - | - | - |
| Post-EO vs Pre-EO | PLE_lf_ | Negative | 1 | - | - | - |
| Post-EC vs Pre-EC | PLE_lf_ | Negative | 1 | - | - | - |
| Pre-EC vs Pre-EO | PLE_hf_ | Positive | 0.013* | 31.487 | 0.004 | 0.007 |
| Post-EC vs Post-EO | PLE_hf_ | Positive | 0.010* | 29.599 | 0.003 | 0.006 |
| Post-EO vs Pre-EO | PLE_hf_ | Negative | 0.001* | -139.878 | 0.001 | 0.002 |
| Post-EC vs Pre-EC | PLE_hf_ | Negative | 0.001* | -127.156 | 0.001 | 0.002 |
| Pre-EC vs Pre-EO | PLE_β_ | Positive | 0.001* | 119.176 | 0.001 | 0.002 |
| Post-EC vs Post-EO | PLE_β_ | Positive | 0.003* | 76.256 | 0.002 | 0.003 |
| Post-EO vs Pre-EO | PLE_β_ | Negative | 0.001* | -179.707 | 0.001 | 0.002 |
| Post-EC vs Pre-EC | PLE_β_ | Negative | 0.001* | -201.745 | 0.001 | 0.002 |

Table S5: Pre- vs post-ketamine Power-law exponent (PLE). *Cluster-based permutation test with α-level = 0.025 for each cluster (total α-level = 0.05).

| Metric | Comparison | Cluster | p value | Cluster-Stat | Std-Dev | CI-Range |
| --- | --- | --- | --- | --- | --- | --- |
| Complexity | Pre-EC vs Pre-EO | Negative | 0.001* | -199.636 | 0.001 | 0.002 |
| Complexity | Post-EC vs Post-EO | Negative | 0.001* | -137.727 | 0.001 | 0.002 |
| Complexity | Post-EO vs Pre-EO | Positive | 0.001* | 165.684 | 0.001 | 0.002 |
| Complexity | Post-EC vs Pre-EC | Positive | 0.001* | 203.351 | 0.001 | 0.002 |
| Entropy | Pre-EC vs Pre-EO | Negative | 0.001* | -199.636 | 0.001 | 0.002 |
| Entropy | Post-EC vs Post-EO | Negative | 0.001* | -137.727 | 0.001 | 0.002 |
| Entropy | Post-EO vs Pre-EO | Positive | 0.001* | 165.684 | 0.001 | 0.002 |
| Entropy | Post-EC vs Pre-EC | Positive | 0.001* | 203.351 | 0.001 | 0.002 |

Table S6: Pre- vs post-ketamine Lempel-Ziv complexity (LZc). Sampling rate 500 Hz, Frequency 1-80 Hz. *Cluster-based permutation test with α-level = 0.025 for each cluster (total α-level = 0.05).

| Metric | Comparison | Cluster | p value | Cluster-Stat | Std-Dev | CI-Range |
| --- | --- | --- | --- | --- | --- | --- |
| Complexity | Pre-EC vs Pre-EO | Negative | 0.001* | -209.294 | 0.001 | 0.002 |
| Complexity | Post-EC vs Post-EO | Negative | 0.001* | -131.909 | 0.001 | 0.002 |
| Complexity | Post-EO vs Pre-EO | Positive | 0.001* | 155.632 | 0.001 | 0.002 |
| Complexity | Post-EC vs Pre-EC | Positive | 0.001* | 208.237 | 0.001 | 0.002 |
| Entropy | Pre-EC vs Pre-EO | Negative | 0.001* | -209.294 | 0.001 | 0.002 |
| Entropy | Post-EC vs Post-EO | Negative | 0.001* | -131.909 | 0.001 | 0.002 |
| Entropy | Post-EO vs Pre-EO | Positive | 0.001* | 155.632 | 0.001 | 0.002 |
| Entropy | Post-EC vs Pre-EC | Positive | 0.001* | 208.237 | 0.001 | 0.002 |

Table S7: Pre- vs post-ketamine Lempel-Ziv complexity (LZc). Sampling rate 160 Hz, Frequency 1-80 Hz. *Cluster-based permutation test with α-level = 0.025 for each cluster (total α-level = 0.05).

| **Metric** | **Comparison** | **Cluster** | **p value** | **Cluster-Stat** | **Std-Dev** | **CI-Range** |
| --- | --- | --- | --- | --- | --- | --- |
| Complexity | Pre-EC vs Pre-EO | Negative | 0.001* | -177.959 | 0.001 | 0.002 |
| Complexity | Post-EC vs Post-EO | Negative | 0.002* | -80.444 | 0.001 | 0.003 |
| Complexity | Post-EO vs Pre-EO | Positive | 0.021* | 12.890 | 0.005 | 0.009 |
| Complexity | Post-EC vs Pre-EC | Positive | 0.001* | 115.392 | 0.001 | 0.002 |
| Entropy | Pre-EC vs Pre-EO | Negative | 0.001* | -177.959 | 0.001 | 0.002 |
| Entropy | Post-EC vs Post-EO | Negative | 0.001* | -80.444 | 0.001 | 0.002 |
| Entropy | Post-EO vs Pre-EO | Positive | 0.019* | 12.890 | 0.004 | 0.008 |
| Entropy | Post-EC vs Pre-EC | Positive | 0.001* | 115.392 | 0.001 | 0.002 |

Table S8: Pre- vs post-ketamine Lempel-Ziv complexity (LZc). Sampling rate 500 Hz, Frequency 1-30 Hz. *Cluster-based permutation test with α-level = 0.025 for each cluster (total α-level = 0.05).

| **Metric** | **Comparison** | **Cluster** | **p value** | **Cluster-Stat** | **Std-Dev** | **CI-Range** |
| --- | --- | --- | --- | --- | --- | --- |
| Complexity | Pre-EC vs Pre-EO | Negative | 0.006* | -46.454 | 0.002 | 0.005 |
| Complexity | Post-EC vs Post-EO | None | 1 | - | - | - |
| Complexity | Post-EO vs Pre-EO | Negative | 0.001* | -94.864 | 0.001 | 0.002 |
| Complexity | Post-EC vs Pre-EC | Negative | 0.005* | -37.394 | 0.002 | 0.004 |
| Entropy | Pre-EC vs Pre-EO | Negative | 0.006* | -46.454 | 0.002 | 0.005 |
| Entropy | Post-EC vs Post-EO | Negative | 1 | - | - | - |
| Entropy | Post-EO vs Pre-EO | Negative | 0.001* | -94.864 | 0.001 | 0.002 |
| Entropy | Post-EC vs Pre-EC | Negative | 0.004* | -37.394 | 0.002 | 0.004 |

Table S9: Pre- vs post-ketamine Lempel-Ziv complexity (LZc). Sampling rate 500 Hz, Frequency 1-20 Hz. *Cluster-based permutation test with α-level = 0.025 for each cluster (total α-level = 0.05).

| **Comparison** | **Frequency** | **β coefficient** | **p value** | **Cohen’s d** |
| --- | --- | --- | --- | --- |
| Pre-EC vs Pre-EO | δ | - 0.01 | < 0.001* | - 0.97 |
| Pre-EC vs Pre-EO | θ | < 0.001 | 0.547 | - 0.11 |
| Pre-EC vs Pre-EO | α | 0.003 | 0.103 | 0.307 |
| Pre-EC vs Pre-EO | β1 | - 0.01 | < 0.001* | - 1.29 |
| Pre-EC vs Pre-EO | high β | - 0.03 | < 0.001* | - 1.58 |
| Pre-EC vs Pre-EO | low γ | - 0.06 | < 0.001* | - 1.31 |
| Pre-EC vs Pre-EO | high γ | - 0.10 | < 0.001* | - 1.17 |
| Pre-EC vs Pre-EO | broadband | - 0.22 | < 0.001* | - 1.33 |
| Post-EC vs Post-EO | δ | - 0.004 | 0.002* | - 0.63 |
| Post-EC vs Post-EO | θ | < 0.001 | 0.716 | - 0.07 |
| Post-EC vs Post-EO | α | 0.01 | 0.005* | 0.56 |
| Post-EC vs Post-EO | β1 | - 0.01 | 0.006* | - 0.54 |
| Post-EC vs Post-EO | high β | - 0.01 | < 0.001* | - 0.75 |
| Post-EC vs Post-EO | low γ | - 0.04 | < 0.001* | - 0.88 |
| Post-EC vs Post-EO | high γ | - 0.06 | < 0.001* | - 0.71 |
| Post-EC vs Post-EO | broadband | - 0.12 | < 0.001* | - 0.76 |
| Post-EO vs Pre-EO | δ | 0.003 | 0.005* | 0.55 |
| Post-EO vs Pre-EO | θ | < 0.001 | 0.784 | - 0.05 |
| Post-EO vs Pre-EO | α | - 0.02 | < 0.001* | - 1.14 |
| Post-EO vs Pre-EO | β1 | - 0.01 | < 0.001* | - 1.13 |
| Post-EO vs Pre-EO | high β | 0.02 | < 0.001* | 1.65 |
| Post-EO vs Pre-EO | low γ | 0.08 | < 0.001* | 1.24 |
| Post-EO vs Pre-EO | high γ | 0.11 | 0.003* | 0.55 |
| Post-EO vs Pre-EO | broadband | 0.21 | <0.001 | 1.29 |
| Post-EC vs Pre-EC | δ | 0.01 | < 0.001* | 0.72 |
| Post-EC vs Pre-EC | θ | < 0.001 | 0.913 | - 0.02 |
| Post-EC vs Pre-EC | α | -0.01 | <0.001* | - 0.93 |
| Post-EC vs Pre-EC | β1 | - 0.003 | 0.208 | - 0.24 |
| Post-EC vs Pre-EC | high β | 0.03 | < 0.001* | 1.62 |
| Post-EC vs Pre-EC | low γ | 0.10 | < 0.001* | 1.79 |
| Post-EC vs Pre-EC | high γ | 0.15 | < 0.001* | 1.37 |
| Post-EC vs Pre-EC | broadband | 0.30 | < 0.001* | 1.55 |

Table S10: Pre- vs post-ketamine Complexity via State-space Entropy Rate (CSER). Sampling rate 160 Hz, Frequency 1-80 Hz. *Linear mixed-effect model with α-level = 0.05.

Correlation between EEG metrics and dose

| Condition | Variable 1 | Variable 2 | β coefficient | p value | p adj |
| --- | --- | --- | --- | --- | --- |
| Pre-EO vs Post-EO | Dose | LZc entropy | 0.17 | 0.369 | 0.820 |
|  | Dose | δ | 0.10 | 0.581 | 0.962 |
|  | Dose | θ | -0.01 | 0.961 | 0.978 |
|  | Dose | α | -0.25 | 0.187 | 0.534 |
|  | Dose | low β | -0.23 | 0.216 | 0.539 |
|  | Dose | high β | -0.13 | 0.484 | 0.962 |
|  | Dose | broadband | -0.10 | 0.587 | 0.962 |
|  | Dose | oscillatory α | -0.06 | 0.758 | 0.978 |
|  | Dose | oscillatory low β | -0.40 | 0.029* | 0.191 |
|  | Dose | oscillatory low γ | 0.27 | 0.156 | 0.520 |
|  | Dose | oscillatory broadband | -0.03 | 0.893 | 0.978 |
|  | Dose | PLE_β_ | -0.35 | 0.056 | 0.260 |
|  | Dose | CSER broadband | -0.01 | 0.953 | 0.978 |
|  | Dose | CSER δ | 0.34 | 0.065 | 0.260 |
|  | Dose | CSER θ | 0.01 | 0.955 | 0.978 |
|  | Dose | CSER α | -0.51 | 0.004 | 0.086 |
|  | Dose | CSER low β | -0.40 | 0.027* | 0.191 |
|  | Dose | CSER high β | 0.01 | 0.978 | 0.978 |
|  | Dose | CSER low γ | 0.09 | 0.625 | 0.962 |
|  | Dose | CSER high γ | 0.06 | 0.745 | 0.978 |
| Pre-EC vs Post-EC | Dose | LZc entropy | 0.33 | 0.071 | 0.264 |
|  | Dose | δ | 0.01 | 0.944 | 0.944 |
|  | Dose | θ | -0.13 | 0.508 | 0.562 |
|  | Dose | α | -0.40 | 0.029 | 0.219 |
|  | Dose | low β | -0.39 | 0.031* | 0.219 |
|  | Dose | high β | -0.32 | 0.083 | 0.264 |
|  | Dose | broadband | -0.23 | 0.230 | 0.291 |
|  | Dose | oscillatory θ | 0.18 | 0.354 | 0.413 |
|  | Dose | oscillatory α | -0.28 | 0.138 | 0.264 |
|  | Dose | oscillatory low β | -0.27 | 0.151 | 0.264 |
|  | Dose | oscillatory low γ | 0.22 | 0.236 | 0.291 |
|  | Dose | oscillatory broadband | -0.25 | 0.174 | 0.264 |
|  | Dose | PLE_β_ | -0.37 | 0.047* | 0.248 |
|  | Dose | CSER broadband | 0.25 | 0.174 | 0.264 |
|  | Dose | CSER δ | 0.27 | 0.147 | 0.264 |
|  | Dose | CSER θ | 0.01 | 0.944 | 0.944 |
|  | Dose | CSER α | -0.41 | 0.023* | 0.219 |
|  | Dose | CSER low β | -0.27 | 0.147 | 0.264 |
|  | Dose | CSER high β | 0.25 | 0.176 | 0.264 |
|  | Dose | CSER low γ | 0.31 | 0.101 | 0.264 |
|  | Dose | CSER high γ | 0.25 | 0.190 | 0.266 |

Table S11: Correlation between EEG metrics raw change and dose. *Spearman correlation test (ρ) with α-level = 0.05.

| Condition | Variable 1 | Variable 2 | Ρ coefficient | p value | p adj |
| --- | --- | --- | --- | --- | --- |
| Pre-EO vs Post-EO | Dose | δ | -0.02 | 0.929 | 0.959 |
|  | Dose | θ | -0.12 | 0.531 | 0.939 |
|  | Dose | α | -0.31 | 0.092 | 0.299 |
|  | Dose | low β | -0.34 | 0.068 | 0.292 |
|  | Dose | high β | -0.08 | 0.688 | 0.939 |
|  | Dose | broadband | -0.03 | 0.868 | 0.959 |
|  | Dose | LZc entropy | 0.1 | 0.603 | 0.939 |
|  | Dose | oscillatory α | -0.08 | 0.655 | 0.939 |
|  | Dose | oscillatory low β | -0.2 | 0.285 | 0.633 |
|  | Dose | oscillatory low γ | 0.3 | 0.105 | 0.299 |
|  | Dose | oscillatory broadband | 0.11 | 0.551 | 0.939 |
|  | Dose | PLE_β_ | -0.36 | 0.052 | 0.292 |
|  | Dose | CSER broadband | -0.07 | 0.705 | 0.939 |
|  | Dose | CSER δ | 0.33 | 0.073 | 0.292 |
|  | Dose | CSER θ | 0.01 | 0.939 | 0.959 |
|  | Dose | CSER α | -0.49 | 0.006* | 0.113 |
|  | Dose | CSER low β | -0.39 | 0.031* | 0.292 |
|  | Dose | CSER high β | -0.06 | 0.754 | 0.942 |
|  | Dose | CSER low γ | 0.01 | 0.959 | 0.959 |
|  | Dose | CSER high γ | 0.28 | 0.129 | 0.322 |
| Pre-EC vs Post-EC | Dose | δ | -0.09 | 0.628 | 0.733 |
|  | Dose | θ | -0.28 | 0.140 | 0.287 |
|  | Dose | α | -0.41 | 0.024* | 0.192 |
|  | Dose | low β | -0.36 | 0.049* | 0.205 |
|  | Dose | high β | -0.22 | 0.239 | 0.358 |
|  | Dose | broadband | -0.01 | 0.967 | 0.967 |
|  | Dose | LZc entropy | 0.3 | 0.102 | 0.271 |
|  | Dose | oscillatory θ | -0.4 | 0.027* | 0.192 |
|  | Dose | oscillatory α | -0.31 | 0.092 | 0.271 |
|  | Dose | oscillatory low β | -0.11 | 0.568 | 0.702 |
|  | Dose | oscillatory low γ | -0.08 | 0.674 | 0.745 |
|  | Dose | oscillatory broadband | -0.38 | 0.040* | 0.205 |
|  | Dose | PLE_β_ | -0.3 | 0.103 | 0.271 |
|  | Dose | CSER broadband | 0.26 | 0.164 | 0.287 |
|  | Dose | CSER δ | 0.27 | 0.150 | 0.287 |
|  | Dose | CSER θ | 0.03 | 0.855 | 0.898 |
|  | Dose | CSER α | -0.41 | 0.024* | 0.192 |
|  | Dose | CSER low β | -0.27 | 0.153 | 0.287 |
|  | Dose | CSER high β | 0.24 | 0.204 | 0.329 |
|  | Dose | CSER low γ | 0.12 | 0.527 | 0.692 |
|  | Dose | CSER high γ | -0.16 | 0.385 | 0.539 |

Table S12: Correlation between EEG metrics relative change and dose. *Spearman correlation test (ρ) with α-level = 0.05.

Correlation between EEG metrics and MADRS at T2

| Condition | Variable 1 | Variable 2 | Ρ coefficient | p value | p adj |
| --- | --- | --- | --- | --- | --- |
| Pre-EO vs Post-EO | MADRS T2 | LZc entropy | -0.05 | 0.791 | 0.879 |
|  | MADRS T2 | δ | -0.08 | 0.666 | 0.858 |
|  | MADRS T2 | θ | -0.37 | 0.046* | 0.562 |
|  | MADRS T2 | α | -0.3 | 0.105 | 0.601 |
|  | MADRS T2 | low β | -0.26 | 0.169 | 0.676 |
|  | MADRS T2 | high β | -0.2 | 0.279 | 0.697 |
|  | MADRS T2 | broadband | -0.29 | 0.120 | 0.601 |
|  | MADRS T2 | oscillatory α | -0.22 | 0.253 | 0.697 |
|  | MADRS T2 | oscillatory low β | 0.07 | 0.729 | 0.858 |
|  | MADRS T2 | oscillatory low γ | -0.35 | 0.056 | 0.562 |
|  | MADRS T2 | oscillatory broadband | -0.23 | 0.221 | 0.697 |
|  | MADRS T2 | PLE_β_ | 0.03 | 0.863 | 0.896 |
|  | MADRS T2 | CSER broadband | -0.12 | 0.513 | 0.835 |
|  | MADRS T2 | CSER δ | 0.07 | 0.727 | 0.858 |
|  | MADRS T2 | CSER θ | -0.08 | 0.659 | 0.858 |
|  | MADRS T2 | CSER α | 0.13 | 0.508 | 0.835 |
|  | MADRS T2 | CSER low β | 0.13 | 0.503 | 0.835 |
|  | MADRS T2 | CSER high β | -0.03 | 0.896 | 0.896 |
|  | MADRS T2 | CSER low γ | -0.15 | 0.421 | 0.835 |
|  | MADRS T2 | CSER high γ | -0.12 | 0.543 | 0.835 |
| Pre-EC vs Post-EC | MADRS T2 | LZc entropy | -0.19 | 0.315 | 0.550 |
|  | MADRS T2 | δ | -0.26 | 0.161 | 0.412 |
|  | MADRS T2 | θ | -0.27 | 0.145 | 0.412 |
|  | MADRS T2 | α | -0.05 | 0.774 | 0.776 |
|  | MADRS T2 | low β | -0.12 | 0.516 | 0.677 |
|  | MADRS T2 | high β | -0.16 | 0.400 | 0.600 |
|  | MADRS T2 | broadband | -0.22 | 0.253 | 0.517 |
|  | MADRS T2 | oscillatory θ | -0.25 | 0.176 | 0.412 |
|  | MADRS T2 | oscillatory α | 0.07 | 0.703 | 0.776 |
|  | MADRS T2 | oscillatory low β | -0.09 | 0.640 | 0.750 |
|  | MADRS T2 | oscillatory low γ | -0.29 | 0.121 | 0.412 |
|  | MADRS T2 | oscillatory broadband | -0.09 | 0.643 | 0.750 |
|  | MADRS T2 | PLE_β_ | 0.26 | 0.169 | 0.412 |
|  | MADRS T2 | CSER broadband | -0.38 | 0.041* | 0.412 |
|  | MADRS T2 | CSER δ | -0.13 | 0.495 | 0.677 |
|  | MADRS T2 | CSER θ | -0.16 | 0.397 | 0.600 |
|  | MADRS T2 | CSER α | 0.29 | 0.124 | 0.412 |
|  | MADRS T2 | CSER low β | 0.05 | 0.776 | 0.776 |
|  | MADRS T2 | CSER high β | -0.21 | 0.271 | 0.517 |
|  | MADRS T2 | CSER low γ | -0.34 | 0.066 | 0.412 |
|  | MADRS T2 | CSER high γ | -0.38 | 0.038 | 0.412 |

Table S13: Correlation between EEG metrics raw change and MADRS at T2. *Spearman correlation test (ρ) with α-level = 0.05.

| Condition | Variable 1 | Variable 2 | Ρ coefficient | p value | p adj |
| --- | --- | --- | --- | --- | --- |
| Pre-EO vs Post-EO | MADRS T2 | δ | -0.18 | 0.330 | 0.939 |
|  | MADRS T2 | θ | -0.32 | 0.089 | 0.939 |
|  | MADRS T2 | α | -0.2 | 0.284 | 0.939 |
|  | MADRS T2 | low β | -0.17 | 0.382 | 0.939 |
|  | MADRS T2 | high β | -0.13 | 0.478 | 0.939 |
|  | MADRS T2 | broadband | -0.21 | 0.261 | 0.939 |
|  | MADRS T2 | LZc entropy | -0.07 | 0.717 | 0.939 |
|  | MADRS T2 | oscillatory α | -0.08 | 0.655 | 0.939 |
|  | MADRS T2 | oscillatory low β | -0.01 | 0.967 | 0.967 |
|  | MADRS T2 | oscillatory low γ | 0.03 | 0.869 | 0.960 |
|  | MADRS T2 | oscillatory broadband | -0.12 | 0.533 | 0.939 |
|  | MADRS T2 | PLE_β_ | 0.06 | 0.751 | 0.939 |
|  | MADRS T2 | CSER broadband | -0.1 | 0.584 | 0.939 |
|  | MADRS T2 | CSER δ | 0.07 | 0.723 | 0.939 |
|  | MADRS T2 | CSER θ | -0.08 | 0.664 | 0.939 |
|  | MADRS T2 | CSER α | 0.14 | 0.450 | 0.939 |
|  | MADRS T2 | CSER low β | 0.12 | 0.533 | 0.939 |
|  | MADRS T2 | CSER high β | 0.02 | 0.912 | 0.960 |
|  | MADRS T2 | CSER low γ | -0.1 | 0.585 | 0.939 |
|  | MADRS T2 | CSER high γ | 0.02 | 0.899 | 0.960 |
| Pre-EC vs Post-EC | MADRS T2 | δ | -0.27 | 0.156 | 0.535 |
|  | MADRS T2 | θ | -0.2 | 0.289 | 0.535 |
|  | MADRS T2 | α | -0.04 | 0.836 | 0.878 |
|  | MADRS T2 | low β | -0.08 | 0.672 | 0.831 |
|  | MADRS T2 | high β | -0.2 | 0.278 | 0.535 |
|  | MADRS T2 | broadband | -0.31 | 0.101 | 0.535 |
|  | MADRS T2 | LZc entropy | -0.2 | 0.280 | 0.535 |
|  | MADRS T2 | oscillatory θ | -0.03 | 0.895 | 0.895 |
|  | MADRS T2 | oscillatory α | 0.06 | 0.757 | 0.878 |
|  | MADRS T2 | oscillatory low β | -0.18 | 0.330 | 0.535 |
|  | MADRS T2 | oscillatory low γ | -0.18 | 0.341 | 0.535 |
|  | MADRS T2 | oscillatory broadband | -0.24 | 0.192 | 0.535 |
|  | MADRS T2 | PLE_β_ | 0.17 | 0.357 | 0.535 |
|  | MADRS T2 | CSER broadband | -0.45 | 0.012* | 0.260 |
|  | MADRS T2 | CSER δ | -0.13 | 0.480 | 0.673 |
|  | MADRS T2 | CSER θ | -0.12 | 0.533 | 0.699 |
|  | MADRS T2 | CSER α | 0.25 | 0.180 | 0.535 |
|  | MADRS T2 | CSER low β | 0.04 | 0.819 | 0.878 |
|  | MADRS T2 | CSER high β | -0.18 | 0.343 | 0.535 |
|  | MADRS T2 | CSER low γ | -0.19 | 0.307 | 0.535 |
|  | MADRS T2 | CSER high γ | 0.38 | 0.036 | 0.379 |

Table S14: Correlation between EEG metrics relative change and MADRS at T2. *Spearman correlation test (ρ) with α-level = 0.05.

Correlation between EEG metrics and CADSS

| Condition | Variable 1 | Variable 2 | Ρ coefficient | p value | p adj |
| --- | --- | --- | --- | --- | --- |
| Pre-EO vs Post-EO | CADSS Broadband | LZc entropy | -0.09 | 0.640 | 0.853 |
|  | CADSS Broadband | δ | 0.34 | 0.068 | 0.276 |
|  | CADSS Broadband | θ | 0.36 | 0.057 | 0.276 |
|  | CADSS Broadband | α | 0.34 | 0.069 | 0.276 |
|  | CADSS Broadband | low β | 0.12 | 0.522 | 0.802 |
|  | CADSS Broadband | high β | 0.22 | 0.262 | 0.655 |
|  | CADSS Broadband | broadband | 0.53 | 0.003* | 0.058 |
|  | CADSS Broadband | oscillatory α | 0.31 | 0.098 | 0.325 |
|  | CADSS Broadband | oscillatory low β | -0.03 | 0.882 | 0.928 |
|  | CADSS Broadband | oscillatory low γ | -0.1 | 0.607 | 0.853 |
|  | CADSS Broadband | oscillatory broadband | 0.46 | 0.011* | 0.112 |
|  | CADSS Broadband | PLE_β_ | -0.15 | 0.437 | 0.728 |
|  | CADSS Broadband | CSER broadband | -0.05 | 0.791 | 0.928 |
|  | CADSS Broadband | CSER δ | -0.15 | 0.435 | 0.728 |
|  | CADSS Broadband | CSER θ | 0.05 | 0.795 | 0.928 |
|  | CADSS Broadband | CSER α | -0.18 | 0.357 | 0.718 |
|  | CADSS Broadband | CSER low β | -0.25 | 0.190 | 0.543 |
|  | CADSS Broadband | CSER high β | -0.18 | 0.359 | 0.718 |
|  | CADSS Broadband | CSER low γ | 0.01 | 0.963 | 0.963 |
|  | CADSS Broadband | CSER high γ | 0.03 | 0.860 | 0.928 |
|  | CADSS Derealization | LZc entropy | 0.02 | 0.921 | 0.946 |
|  | CADSS Derealization | δ | 0.3 | 0.114 | 0.438 |
|  | CADSS Derealization | θ | 0.41 | 0.027* | 0.152 |
|  | CADSS Derealization | α | 0.4 | 0.030* | 0.152 |
|  | CADSS Derealization | low β | 0.25 | 0.197 | 0.438 |
|  | CADSS Derealization | high β | 0.29 | 0.132 | 0.438 |
|  | CADSS Derealization | broadband | 0.69 | < 0.001* | 0.001* |
|  | CADSS Derealization | oscillatory α | 0.27 | 0.157 | 0.438 |
|  | CADSS Derealization | oscillatory low β | 0.05 | 0.784 | 0.943 |
|  | CADSS Derealization | oscillatory low γ | 0.01 | 0.946 | 0.946 |
|  | CADSS Derealization | oscillatory broadband | 0.42 | 0.024* | 0.152 |
|  | CADSS Derealization | PLE_β_ | -0.09 | 0.634 | 0.906 |
|  | CADSS Derealization | CSER broadband | 0.08 | 0.690 | 0.919 |
|  | CADSS Derealization | CSER δ | -0.26 | 0.182 | 0.438 |
|  | CADSS Derealization | CSER θ | 0.05 | 0.801 | 0.943 |
|  | CADSS Derealization | CSER α | -0.02 | 0.924 | 0.946 |
|  | CADSS Derealization | CSER low β | -0.17 | 0.366 | 0.666 |
|  | CADSS Derealization | CSER high β | -0.15 | 0.448 | 0.747 |
|  | CADSS Derealization | CSER low γ | 0.12 | 0.548 | 0.844 |
|  | CADSS Derealization | CSER high γ | 0.18 | 0.361 | 0.666 |
|  | CADSS Derealization | LZc entropy | 0.02 | 0.921 | 0.946 |
|  | CADSS Depersonalization | LZc entropy | 0.08 | 0.679 | 0.948 |
|  | CADSS Depersonalization | δ | 0.21 | 0.276 | 0.848 |
|  | CADSS Depersonalization | θ | 0.16 | 0.422 | 0.848 |
|  | CADSS Depersonalization | α | 0.15 | 0.424 | 0.848 |
|  | CADSS Depersonalization | low β | 0.02 | 0.898 | 0.948 |
|  | CADSS Depersonalization | high β | 0.17 | 0.381 | 0.848 |
|  | CADSS Depersonalization | broadband | 0.3 | 0.113 | 0.796 |
|  | CADSS Depersonalization | oscillatory α | 0.19 | 0.336 | 0.848 |
|  | CADSS Depersonalization | oscillatory low β | 0.02 | 0.915 | 0.948 |
|  | CADSS Depersonalization | oscillatory low γ | -0.01 | 0.948 | 0.948 |
|  | CADSS Depersonalization | oscillatory broadband | 0.35 | 0.063 | 0.796 |
|  | CADSS Depersonalization | PLE_β_ | -0.3 | 0.119 | 0.796 |
|  | CADSS Depersonalization | CSER broadband | 0.09 | 0.652 | 0.948 |
|  | CADSS Depersonalization | CSER δ | -0.03 | 0.874 | 0.948 |
|  | CADSS Depersonalization | CSER θ | -0.14 | 0.474 | 0.862 |
|  | CADSS Depersonalization | CSER α | -0.26 | 0.181 | 0.848 |
|  | CADSS Depersonalization | CSER low β | -0.07 | 0.733 | 0.948 |
|  | CADSS Depersonalization | CSER high β | 0.03 | 0.866 | 0.948 |
|  | CADSS Depersonalization | CSER low γ | 0.16 | 0.406 | 0.848 |
|  | CADSS Depersonalization | CSER high γ | 0.11 | 0.572 | 0.948 |
|  | CADSS Depersonalization | LZc entropy | 0.08 | 0.679 | 0.948 |
|  | CADSS Amnesia | LZc entropy | -0.1 | 0.591 | 0.927 |
|  | CADSS Amnesia | δ | 0.11 | 0.560 | 0.927 |
|  | CADSS Amnesia | θ | -0.09 | 0.654 | 0.927 |
|  | CADSS Amnesia | α | -0.04 | 0.835 | 0.927 |
|  | CADSS Amnesia | low β | -0.2 | 0.290 | 0.927 |
|  | CADSS Amnesia | high β | 0.02 | 0.927 | 0.927 |
|  | CADSS Amnesia | broadband | 0.09 | 0.641 | 0.927 |
|  | CADSS Amnesia | oscillatory α | 0.1 | 0.599 | 0.927 |
|  | CADSS Amnesia | oscillatory low β | -0.22 | 0.259 | 0.927 |
|  | CADSS Amnesia | oscillatory low γ | -0.26 | 0.177 | 0.927 |
|  | CADSS Amnesia | oscillatory broadband | 0.14 | 0.463 | 0.927 |
|  | CADSS Amnesia | PLE_β_ | -0.04 | 0.830 | 0.927 |
|  | CADSS Amnesia | CSER broadband | -0.07 | 0.700 | 0.927 |
|  | CADSS Amnesia | CSER δ | -0.13 | 0.488 | 0.927 |
|  | CADSS Amnesia | CSER θ | -0.11 | 0.568 | 0.927 |
|  | CADSS Amnesia | CSER α | -0.13 | 0.494 | 0.927 |
|  | CADSS Amnesia | CSER low β | -0.13 | 0.515 | 0.927 |
|  | CADSS Amnesia | CSER high β | -0.09 | 0.652 | 0.927 |
|  | CADSS Amnesia | CSER low γ | -0.04 | 0.831 | 0.927 |
|  | CADSS Amnesia | CSER high γ | -0.02 | 0.913 | 0.927 |
|  | CADSS Amnesia | LZc entropy | -0.1 | 0.591 | 0.927 |
| Pre-EC vs Post-EC | CADSS Broadband | LZc entropy | 0.03 | 0.897 | 0.942 |
|  | CADSS Broadband | δ | 0.46 | 0.011* | 0.239 |
|  | CADSS Broadband | θ | 0.34 | 0.071 | 0.298 |
|  | CADSS Broadband | α | 0.14 | 0.481 | 0.917 |
|  | CADSS Broadband | low β | 0.06 | 0.759 | 0.917 |
|  | CADSS Broadband | high β | 0.22 | 0.243 | 0.638 |
|  | CADSS Broadband | broadband | 0.39 | 0.036* | 0.255 |
|  | CADSS Broadband | oscillatory θ | 0.28 | 0.136 | 0.478 |
|  | CADSS Broadband | oscillatory α | 0.24 | 0.216 | 0.638 |
|  | CADSS Broadband | oscillatory low β | -0.16 | 0.422 | 0.886 |
|  | CADSS Broadband | oscillatory low γ | -0.05 | 0.816 | 0.917 |
|  | CADSS Broadband | oscillatory broadband | 0.4 | 0.031* | 0.255 |
|  | CADSS Broadband | PLE_β_ | -0.09 | 0.637 | 0.917 |
|  | CADSS Broadband | CSER broadband | -0.05 | 0.797 | 0.917 |
|  | CADSS Broadband | CSER δ | 0.12 | 0.537 | 0.917 |
|  | CADSS Broadband | CSER θ | 0.07 | 0.729 | 0.917 |
|  | CADSS Broadband | CSER α | -0.35 | 0.064 | 0.298 |
|  | CADSS Broadband | CSER low β | -0.19 | 0.328 | 0.766 |
|  | CADSS Broadband | CSER high β | -0.04 | 0.830 | 0.917 |
|  | CADSS Broadband | CSER low γ | -0.05 | 0.798 | 0.917 |
|  | CADSS Broadband | CSER high γ | 0.01 | 0.958 | 0.958 |
|  | CADSS Derealization | LZc entropy | -0.04 | 0.850 | 0.963 |
|  | CADSS Derealization | δ | 0.53 | 0.003* | 0.070 |
|  | CADSS Derealization | θ | 0.41 | 0.028* | 0.182 |
|  | CADSS Derealization | α | 0.19 | 0.331 | 0.696 |
|  | CADSS Derealization | low β | 0.08 | 0.666 | 0.963 |
|  | CADSS Derealization | high β | 0.29 | 0.131 | 0.459 |
|  | CADSS Derealization | broadband | 0.47 | 0.010* | 0.100 |
|  | CADSS Derealization | oscillatory θ | 0.37 | 0.051 | 0.214 |
|  | CADSS Derealization | oscillatory α | 0.19 | 0.317 | 0.696 |
|  | CADSS Derealization | oscillatory low β | -0.16 | 0.406 | 0.775 |
|  | CADSS Derealization | oscillatory low γ | 0.01 | 0.963 | 0.963 |
|  | CADSS Derealization | oscillatory broadband | 0.39 | 0.035* | 0.182 |
|  | CADSS Derealization | PLE_β_ | -0.05 | 0.791 | 0.963 |
|  | CADSS Derealization | CSER broadband | -0.02 | 0.936 | 0.963 |
|  | CADSS Derealization | CSER δ | 0.13 | 0.516 | 0.903 |
|  | CADSS Derealization | CSER θ | 0.05 | 0.793 | 0.963 |
|  | CADSS Derealization | CSER α | -0.19 | 0.326 | 0.696 |
|  | CADSS Derealization | CSER low β | -0.22 | 0.245 | 0.696 |
|  | CADSS Derealization | CSER high β | -0.05 | 0.781 | 0.963 |
|  | CADSS Derealization | CSER low γ | -0.02 | 0.931 | 0.963 |
|  | CADSS Derealization | CSER high γ | 0.03 | 0.896 | 0.963 |
|  | CADSS Depersonalization | LZc entropy | 0.13 | 0.514 | 0.934 |
|  | CADSS Depersonalization | δ | 0.23 | 0.230 | 0.934 |
|  | CADSS Depersonalization | θ | 0.13 | 0.486 | 0.934 |
|  | CADSS Depersonalization | α | -0.03 | 0.863 | 0.934 |
|  | CADSS Depersonalization | low β | -0.04 | 0.844 | 0.934 |
|  | CADSS Depersonalization | high β | 0.14 | 0.483 | 0.934 |
|  | CADSS Depersonalization | broadband | 0.14 | 0.455 | 0.934 |
|  | CADSS Depersonalization | oscillatory θ | 0.08 | 0.674 | 0.934 |
|  | CADSS Depersonalization | oscillatory α | 0.1 | 0.609 | 0.934 |
|  | CADSS Depersonalization | oscillatory low β | -0.01 | 0.944 | 0.944 |
|  | CADSS Depersonalization | oscillatory low γ | -0.03 | 0.890 | 0.934 |
|  | CADSS Depersonalization | oscillatory broadband | 0.19 | 0.311 | 0.934 |
|  | CADSS Depersonalization | PLE_β_ | -0.08 | 0.693 | 0.934 |
|  | CADSS Depersonalization | CSER broadband | 0.05 | 0.789 | 0.934 |
|  | CADSS Depersonalization | CSER δ | 0.19 | 0.312 | 0.934 |
|  | CADSS Depersonalization | CSER θ | -0.03 | 0.889 | 0.934 |
|  | CADSS Depersonalization | CSER α | -0.29 | 0.130 | 0.934 |
|  | CADSS Depersonalization | CSER low β | 0.03 | 0.871 | 0.934 |
|  | CADSS Depersonalization | CSER high β | 0.18 | 0.349 | 0.934 |
|  | CADSS Depersonalization | CSER low γ | 0.05 | 0.817 | 0.934 |
|  | CADSS Depersonalization | CSER high γ | 0.06 | 0.773 | 0.934 |
|  | CADSS Amnesia | LZc entropy | -0.17 | 0.391 | 0.988 |
|  | CADSS Amnesia | δ | 0.01 | 0.961 | 0.988 |
|  | CADSS Amnesia | θ | -0.07 | 0.708 | 0.988 |
|  | CADSS Amnesia | α | -0.03 | 0.872 | 0.988 |
|  | CADSS Amnesia | low β | -0.12 | 0.543 | 0.988 |
|  | CADSS Amnesia | high β | 0.03 | 0.877 | 0.988 |
|  | CADSS Amnesia | broadband | -0.06 | 0.751 | 0.988 |
|  | CADSS Amnesia | oscillatory θ | -0.07 | 0.712 | 0.988 |
|  | CADSS Amnesia | oscillatory α | 0.05 | 0.787 | 0.988 |
|  | CADSS Amnesia | oscillatory low β | -0.16 | 0.413 | 0.988 |
|  | CADSS Amnesia | oscillatory low γ | -0.34 | 0.073 | 0.988 |
|  | CADSS Amnesia | oscillatory broadband | 0 | 0.988 | 0.988 |
|  | CADSS Amnesia | PLE_β_ | 0.21 | 0.266 | 0.988 |
|  | CADSS Amnesia | CSER broadband | -0.27 | 0.149 | 0.988 |
|  | CADSS Amnesia | CSER δ | -0.02 | 0.899 | 0.988 |
|  | CADSS Amnesia | CSER θ | -0.04 | 0.839 | 0.988 |
|  | CADSS Amnesia | CSER α | 0.01 | 0.973 | 0.988 |
|  | CADSS Amnesia | CSER low β | -0.07 | 0.713 | 0.988 |
|  | CADSS Amnesia | CSER high β | -0.15 | 0.426 | 0.988 |
|  | CADSS Amnesia | CSER low γ | -0.31 | 0.104 | 0.988 |
|  | CADSS Amnesia | CSER high γ | -0.2 | 0.294 | 0.988 |

Table S15: Correlation between EEG metrics raw change and CADSS (N = 29; no outlier). *Spearman correlation test (ρ) with α-level = 0.05.

| Condition | Variable 1 | Variable 2 | Ρ coefficient | p value | p adj |
| --- | --- | --- | --- | --- | --- |
| Pre-EO vs Post-EO | CADSS Broadband | δ | 0.29 | 0.131 | 0.627 |
|  | CADSS Broadband | θ | 0.29 | 0.125 | 0.627 |
|  | CADSS Broadband | α | 0.17 | 0.376 | 0.627 |
|  | CADSS Broadband | low β | 0.18 | 0.344 | 0.627 |
|  | CADSS Broadband | high β | 0.24 | 0.218 | 0.627 |
|  | CADSS Broadband | broadband | 0.48 | 0.009* | 0.175 |
|  | CADSS Broadband | LZc entropy | -0.11 | 0.561 | 0.802 |
|  | CADSS Broadband | oscillatory α | 0.06 | 0.763 | 0.846 |
|  | CADSS Broadband | oscillatory low β | -0.25 | 0.200 | 0.627 |
|  | CADSS Broadband | oscillatory low γ | -0.2 | 0.297 | 0.627 |
|  | CADSS Broadband | oscillatory broadband | 0.05 | 0.803 | 0.846 |
|  | CADSS Broadband | PLE_β_ | -0.19 | 0.316 | 0.627 |
|  | CADSS Broadband | CSER broadband | -0.08 | 0.666 | 0.807 |
|  | CADSS Broadband | CSER δ | -0.15 | 0.428 | 0.658 |
|  | CADSS Broadband | CSER θ | 0.01 | 0.956 | 0.956 |
|  | CADSS Broadband | CSER α | -0.18 | 0.353 | 0.627 |
|  | CADSS Broadband | CSER low β | -0.24 | 0.217 | 0.627 |
|  | CADSS Broadband | CSER high β | -0.22 | 0.258 | 0.627 |
|  | CADSS Broadband | CSER low γ | -0.08 | 0.686 | 0.807 |
|  | CADSS Broadband | CSER high γ | -0.08 | 0.682 | 0.807 |
|  | CADSS Derealization | δ | 0.29 | 0.122 | 0.407 |
|  | CADSS Derealization | θ | 0.36 | 0.057 | 0.382 |
|  | CADSS Derealization | α | 0.32 | 0.096 | 0.407 |
|  | CADSS Derealization | low β | 0.3 | 0.111 | 0.407 |
|  | CADSS Derealization | high β | 0.36 | 0.057 | 0.382 |
|  | CADSS Derealization | broadband | 0.64 | < 0.001* | 0.004* |
|  | CADSS Derealization | LZc entropy | 0.01 | 0.956 | 0.978 |
|  | CADSS Derealization | oscillatory α | 0.13 | 0.510 | 0.851 |
|  | CADSS Derealization | oscillatory low β | -0.01 | 0.978 | 0.978 |
|  | CADSS Derealization | oscillatory low γ | -0.21 | 0.282 | 0.706 |
|  | CADSS Derealization | oscillatory broadband | 0.03 | 0.866 | 0.978 |
|  | CADSS Derealization | PLE_β_ | -0.1 | 0.602 | 0.861 |
|  | CADSS Derealization | CSER broadband | 0.07 | 0.731 | 0.975 |
|  | CADSS Derealization | CSER δ | -0.26 | 0.168 | 0.481 |
|  | CADSS Derealization | CSER θ | 0.01 | 0.953 | 0.978 |
|  | CADSS Derealization | CSER α | -0.01 | 0.964 | 0.978 |
|  | CADSS Derealization | CSER low β | -0.16 | 0.410 | 0.764 |
|  | CADSS Derealization | CSER high β | -0.16 | 0.420 | 0.764 |
|  | CADSS Derealization | CSER low γ | 0.11 | 0.572 | 0.861 |
|  | CADSS Derealization | CSER high γ | -0.16 | 0.399 | 0.764 |
|  | CADSS Derealization | δ | 0.29 | 0.122 | 0.407 |
|  | CADSS Depersonalization | δ | 0.09 | 0.639 | 0.937 |
|  | CADSS Depersonalization | θ | 0.09 | 0.657 | 0.937 |
|  | CADSS Depersonalization | α | -0.05 | 0.811 | 0.937 |
|  | CADSS Depersonalization | low β | 0.05 | 0.812 | 0.937 |
|  | CADSS Depersonalization | high β | 0.1 | 0.624 | 0.937 |
|  | CADSS Depersonalization | broadband | 0.26 | 0.169 | 0.915 |
|  | CADSS Depersonalization | LZc entropy | 0.09 | 0.654 | 0.937 |
|  | CADSS Depersonalization | oscillatory α | -0.18 | 0.349 | 0.937 |
|  | CADSS Depersonalization | oscillatory low β | -0.39 | 0.039* | 0.776 |
|  | CADSS Depersonalization | oscillatory low γ | -0.22 | 0.254 | 0.937 |
|  | CADSS Depersonalization | oscillatory broadband | -0.11 | 0.580 | 0.937 |
|  | CADSS Depersonalization | PLE_β_ | -0.32 | 0.089 | 0.889 |
|  | CADSS Depersonalization | CSER broadband | 0.05 | 0.801 | 0.937 |
|  | CADSS Depersonalization | CSER δ | -0.04 | 0.846 | 0.937 |
|  | CADSS Depersonalization | CSER θ | -0.18 | 0.361 | 0.937 |
|  | CADSS Depersonalization | CSER α | -0.25 | 0.183 | 0.915 |
|  | CADSS Depersonalization | CSER low β | -0.06 | 0.756 | 0.937 |
|  | CADSS Depersonalization | CSER high β | -0.03 | 0.891 | 0.937 |
|  | CADSS Depersonalization | CSER low γ | 0 | 0.991 | 0.991 |
|  | CADSS Depersonalization | CSER high γ | 0.03 | 0.886 | 0.937 |
|  | CADSS Depersonalization | δ | 0.09 | 0.639 | 0.937 |
|  | CADSS Amnesia | δ | -0.02 | 0.927 | 0.931 |
|  | CADSS Amnesia | θ | -0.1 | 0.591 | 0.865 |
|  | CADSS Amnesia | α | -0.08 | 0.670 | 0.865 |
|  | CADSS Amnesia | low β | -0.06 | 0.755 | 0.865 |
|  | CADSS Amnesia | high β | 0.07 | 0.732 | 0.865 |
|  | CADSS Amnesia | broadband | 0.05 | 0.779 | 0.865 |
|  | CADSS Amnesia | LZc entropy | -0.15 | 0.426 | 0.865 |
|  | CADSS Amnesia | oscillatory α | -0.06 | 0.754 | 0.865 |
|  | CADSS Amnesia | oscillatory low β | -0.06 | 0.751 | 0.865 |
|  | CADSS Amnesia | oscillatory low γ | -0.12 | 0.535 | 0.865 |
|  | CADSS Amnesia | oscillatory broadband | 0.11 | 0.580 | 0.865 |
|  | CADSS Amnesia | PLE_β_ | -0.11 | 0.566 | 0.865 |
|  | CADSS Amnesia | CSER broadband | -0.11 | 0.559 | 0.865 |
|  | CADSS Amnesia | CSER δ | -0.13 | 0.516 | 0.865 |
|  | CADSS Amnesia | CSER θ | -0.15 | 0.447 | 0.865 |
|  | CADSS Amnesia | CSER α | -0.13 | 0.512 | 0.865 |
|  | CADSS Amnesia | CSER low β | -0.12 | 0.520 | 0.865 |
|  | CADSS Amnesia | CSER high β | -0.14 | 0.462 | 0.865 |
|  | CADSS Amnesia | CSER low γ | -0.14 | 0.468 | 0.865 |
|  | CADSS Amnesia | CSER high γ | -0.02 | 0.931 | 0.931 |
|  | CADSS Amnesia | δ | -0.02 | 0.927 | 0.931 |
| Pre-EC vs Post-EC | CADSS Broadband | δ | 0.37 | 0.049* | 0.601 |
|  | CADSS Broadband | θ | 0.19 | 0.326 | 0.760 |
|  | CADSS Broadband | α | -0.09 | 0.643 | 0.852 |
|  | CADSS Broadband | low β | -0.02 | 0.938 | 0.950 |
|  | CADSS Broadband | high β | 0.14 | 0.478 | 0.773 |
|  | CADSS Broadband | broadband | 0.19 | 0.318 | 0.760 |
|  | CADSS Broadband | LZc entropy | -0.04 | 0.819 | 0.950 |
|  | CADSS Broadband | oscillatory θ | 0.09 | 0.649 | 0.852 |
|  | CADSS Broadband | oscillatory α | -0.01 | 0.950 | 0.950 |
|  | CADSS Broadband | oscillatory low β | -0.03 | 0.896 | 0.950 |
|  | CADSS Broadband | oscillatory low γ | 0.23 | 0.237 | 0.760 |
|  | CADSS Broadband | oscillatory broadband | 0.25 | 0.199 | 0.760 |
|  | CADSS Broadband | PLE_β_ | -0.26 | 0.180 | 0.760 |
|  | CADSS Broadband | CSER broadband | -0.16 | 0.412 | 0.773 |
|  | CADSS Broadband | CSER δ | 0.15 | 0.450 | 0.773 |
|  | CADSS Broadband | CSER θ | 0.05 | 0.782 | 0.950 |
|  | CADSS Broadband | CSER α | -0.36 | 0.057 | 0.601 |
|  | CADSS Broadband | CSER low β | -0.19 | 0.316 | 0.760 |
|  | CADSS Broadband | CSER high β | -0.24 | 0.212 | 0.760 |
|  | CADSS Broadband | CSER low γ | 0.11 | 0.561 | 0.841 |
|  | CADSS Broadband | CSER high γ | 0.14 | 0.459 | 0.773 |
|  | CADSS Derealization | δ | 0.48 | 0.009* | 0.191 |
|  | CADSS Derealization | θ | 0.26 | 0.167 | 0.702 |
|  | CADSS Derealization | α | 0.07 | 0.702 | 0.846 |
|  | CADSS Derealization | low β | 0.05 | 0.791 | 0.846 |
|  | CADSS Derealization | high β | 0.24 | 0.214 | 0.712 |
|  | CADSS Derealization | broadband | 0.3 | 0.109 | 0.702 |
|  | CADSS Derealization | LZc entropy | -0.07 | 0.724 | 0.846 |
|  | CADSS Derealization | oscillatory θ | 0.28 | 0.149 | 0.702 |
|  | CADSS Derealization | oscillatory α | 0.14 | 0.453 | 0.733 |
|  | CADSS Derealization | oscillatory low β | -0.07 | 0.728 | 0.846 |
|  | CADSS Derealization | oscillatory low γ | 0.29 | 0.129 | 0.702 |
|  | CADSS Derealization | oscillatory broadband | 0.1 | 0.623 | 0.846 |
|  | CADSS Derealization | PLE_β_ | -0.2 | 0.305 | 0.733 |
|  | CADSS Derealization | CSER broadband | -0.08 | 0.691 | 0.846 |
|  | CADSS Derealization | CSER δ | 0.15 | 0.443 | 0.733 |
|  | CADSS Derealization | CSER θ | 0.05 | 0.805 | 0.846 |
|  | CADSS Derealization | CSER α | -0.17 | 0.383 | 0.733 |
|  | CADSS Derealization | CSER low β | -0.23 | 0.237 | 0.712 |
|  | CADSS Derealization | CSER high β | -0.18 | 0.346 | 0.733 |
|  | CADSS Derealization | CSER low γ | 0.16 | 0.413 | 0.733 |
|  | CADSS Derealization | CSER high γ | 0.01 | 0.945 | 0.945 |
|  | CADSS Depersonalization | δ | 0.11 | 0.574 | 0.966 |
|  | CADSS Depersonalization | θ | -0.02 | 0.898 | 0.966 |
|  | CADSS Depersonalization | α | -0.3 | 0.117 | 0.818 |
|  | CADSS Depersonalization | low β | -0.15 | 0.439 | 0.966 |
|  | CADSS Depersonalization | high β | 0.02 | 0.921 | 0.966 |
|  | CADSS Depersonalization | broadband | 0.01 | 0.966 | 0.966 |
|  | CADSS Depersonalization | LZc entropy | 0.08 | 0.679 | 0.966 |
|  | CADSS Depersonalization | oscillatory θ | -0.1 | 0.601 | 0.966 |
|  | CADSS Depersonalization | oscillatory α | -0.12 | 0.521 | 0.966 |
|  | CADSS Depersonalization | oscillatory low β | -0.01 | 0.942 | 0.966 |
|  | CADSS Depersonalization | oscillatory low γ | 0.1 | 0.624 | 0.966 |
|  | CADSS Depersonalization | oscillatory broadband | 0.34 | 0.072 | 0.818 |
|  | CADSS Depersonalization | PLE_β_ | -0.24 | 0.217 | 0.966 |
|  | CADSS Depersonalization | CSER broadband | -0.1 | 0.624 | 0.966 |
|  | CADSS Depersonalization | CSER δ | 0.21 | 0.273 | 0.966 |
|  | CADSS Depersonalization | CSER θ | -0.06 | 0.751 | 0.966 |
|  | CADSS Depersonalization | CSER α | -0.3 | 0.108 | 0.818 |
|  | CADSS Depersonalization | CSER low β | 0.05 | 0.816 | 0.966 |
|  | CADSS Depersonalization | CSER high β | -0.03 | 0.882 | 0.966 |
|  | CADSS Depersonalization | CSER low γ | 0.15 | 0.448 | 0.966 |
|  | CADSS Depersonalization | CSER high γ | -0.06 | 0.771 | 0.966 |
|  | CADSS Amnesia | δ | -0.03 | 0.892 | 0.983 |
|  | CADSS Amnesia | θ | -0.08 | 0.686 | 0.983 |
|  | CADSS Amnesia | α | -0.08 | 0.664 | 0.983 |
|  | CADSS Amnesia | low β | -0.1 | 0.622 | 0.983 |
|  | CADSS Amnesia | high β | -0.05 | 0.811 | 0.983 |
|  | CADSS Amnesia | broadband | -0.17 | 0.374 | 0.983 |
|  | CADSS Amnesia | LZc entropy | -0.24 | 0.212 | 0.840 |
|  | CADSS Amnesia | oscillatory θ | 0.27 | 0.156 | 0.820 |
|  | CADSS Amnesia | oscillatory α | 0.23 | 0.240 | 0.840 |
|  | CADSS Amnesia | oscillatory low β | 0 | 0.983 | 0.983 |
|  | CADSS Amnesia | oscillatory low γ | -0.02 | 0.931 | 0.983 |
|  | CADSS Amnesia | oscillatory broadband | -0.02 | 0.916 | 0.983 |
|  | CADSS Amnesia | PLE_β_ | 0.14 | 0.466 | 0.983 |
|  | CADSS Amnesia | CSER broadband | -0.31 | 0.103 | 0.820 |
|  | CADSS Amnesia | CSER δ | 0.01 | 0.953 | 0.983 |
|  | CADSS Amnesia | CSER θ | -0.04 | 0.848 | 0.983 |
|  | CADSS Amnesia | CSER α | -0.01 | 0.945 | 0.983 |
|  | CADSS Amnesia | CSER low β | -0.06 | 0.757 | 0.983 |
|  | CADSS Amnesia | CSER high β | -0.29 | 0.125 | 0.820 |
|  | CADSS Amnesia | CSER low γ | -0.09 | 0.641 | 0.983 |
|  | CADSS Amnesia | CSER high γ | 0.33 | 0.083 | 0.820 |

Table S16: Correlation between EEG metrics relative change and CADSS (N = 29; no outlier). *Spearman correlation test (ρ) with α-level = 0.05.

Early vs Late responders

| **Time** | **Group** | **EEG Metric** | **n** | **mean** | **sd** |
| --- | --- | --- | --- | --- | --- |
| Pre-EC | Early | PSD δ | 18 | 0.758 | 0.237 |
| Pre-EC | Early | PSD θ | 18 | 0.843 | 0.423 |
| Pre-EC | Early | PSD α | 18 | 1.039 | 0.483 |
| Pre-EC | Early | PSD low β | 18 | 0.464 | 0.169 |
| Pre-EC | Early | PSD high β | 18 | 0.284 | 0.102 |
| Pre-EC | Early | PSD low γ | 18 | 0.171 | 0.058 |
| Pre-EC | Early | PSD high γ | 18 | 0.102 | 0.04 |
| Pre-EC | Early | Broadband | 18 | 0.295 | 0.094 |
| Pre-EC | Early | δ Oscillatory | 18 | 0.079 | 0.073 |
| Pre-EC | Early | δ Fractal | 18 | 0.575 | 0.354 |
| Pre-EC | Early | θ Oscillatory | 18 | 0.334 | 0.648 |
| Pre-EC | Early | θ Fractal | 18 | 0.575 | 0.354 |
| Pre-EC | Early | α Oscillatory | 18 | 1.386 | 1.314 |
| Pre-EC | Early | α Fractal | 18 | 0.422 | 0.26 |
| Pre-EC | Early | low β Oscillatory | 18 | 0.008 | 0.076 |
| Pre-EC | Early | low β Fractal | 18 | 0.252 | 0.151 |
| Pre-EC | Early | high β Oscillatory | 18 | 0.029 | 0.037 |
| Pre-EC | Early | high β Fractal | 18 | 0.073 | 0.039 |
| Pre-EC | Early | low γ Oscillatory | 18 | 0.004 | 0.006 |
| Pre-EC | Early | low γ Fractal | 18 | 0.031 | 0.018 |
| Pre-EC | Early | high γ Oscillatory | 18 | 0.013 | 0.011 |
| Pre-EC | Early | high γ Fractal | 18 | 0.002 | 0.002 |
| Pre-EC | Early | Broadband Oscillatory | 18 | 0.115 | 0.086 |
| Pre-EC | Early | Broadband Fractal | 18 | 0.121 | 0.067 |
| Pre-EC | Early | LZC | 18 | 0.367 | 0.059 |
| Pre-EC | Early | PLE 1-80hz | 18 | 0.113 | 0.01 |
| Pre-EC | Early | PLE 1-20hz | 18 | 0.091 | 0.037 |
| Pre-EC | Early | PLE 20-30hz | 18 | 0.09 | 0.022 |
| Pre-EC | Early | PLE_20-80hz | 18 | 0.116 | 0.01 |
| Pre-EC | Early | CSER Broadband | 18 | 0.572 | 0.235 |
| Pre-EC | Early | CSER δ | 18 | 0.071 | 0.008 |
| Pre-EC | Early | CSER θ | 18 | 0.095 | 0.015 |
| Pre-EC | Early | CSER α | 18 | 0.152 | 0.014 |
| Pre-EC | Early | CSER low β | 18 | 0.106 | 0.014 |
| Pre-EC | Early | CSER high β | 18 | 0.116 | 0.04 |
| Pre-EC | Early | CSER low γ | 18 | 0.088 | 0.069 |
| Pre-EC | Early | CSER high γ | 18 | -0.064 | 0.12 |
| Pre-EC | Late | PSD δ | 12 | 0.685 | 0.208 |
| Pre-EC | Late | PSD θ | 12 | 0.7 | 0.271 |
| Pre-EC | Late | PSD α | 12 | 1.01 | 0.316 |
| Pre-EC | Late | PSD low β | 12 | 0.454 | 0.143 |
| Pre-EC | Late | PSD high β | 12 | 0.248 | 0.063 |
| Pre-EC | Late | PSD low γ | 12 | 0.147 | 0.038 |
| Pre-EC | Late | PSD high γ | 12 | 0.086 | 0.028 |
| Pre-EC | Late | Broadband | 12 | 0.265 | 0.061 |
| Pre-EC | Late | δ Oscillatory | 12 | 0.109 | 0.164 |
| Pre-EC | Late | δ Fractal | 12 | 0.456 | 0.279 |
| Pre-EC | Late | θ Oscillatory | 12 | 0.12 | 0.196 |
| Pre-EC | Late | θ Fractal | 12 | 0.456 | 0.279 |
| Pre-EC | Late | α Oscillatory | 12 | 1.125 | 0.747 |
| Pre-EC | Late | α Fractal | 12 | 0.355 | 0.194 |
| Pre-EC | Late | low β Oscillatory | 12 | 0.031 | 0.059 |
| Pre-EC | Late | low β Fractal | 12 | 0.215 | 0.107 |
| Pre-EC | Late | high β Oscillatory | 12 | 0.019 | 0.022 |
| Pre-EC | Late | high β Fractal | 12 | 0.06 | 0.03 |
| Pre-EC | Late | low γ Oscillatory | 12 | 0.002 | 0.003 |
| Pre-EC | Late | low γ Fractal | 12 | 0.023 | 0.012 |
| Pre-EC | Late | high γ Oscillatory | 12 | 0.009 | 0.006 |
| Pre-EC | Late | high γ Fractal | 12 | 0.001 | 0.001 |
| Pre-EC | Late | Broadband_Oscillatory | 12 | 0.089 | 0.051 |
| Pre-EC | Late | Broadband_Fractal | 12 | 0.099 | 0.045 |
| Pre-EC | Late | LZC | 12 | 0.356 | 0.044 |
| Pre-EC | Late | PLE 1-80hz | 12 | 0.115 | 0.01 |
| Pre-EC | Late | PLE 1-0hz | 12 | 0.084 | 0.035 |
| Pre-EC | Late | PLE 20-30hz | 12 | 0.099 | 0.018 |
| Pre-EC | Late | PLE 20-80hz | 12 | 0.118 | 0.01 |
| Pre-EC | Late | CSER Broadband | 12 | 0.542 | 0.212 |
| Pre-EC | Late | CSER δ | 12 | 0.07 | 0.008 |
| Pre-EC | Late | CSER θ | 12 | 0.092 | 0.01 |
| Pre-EC | Late | CSER α | 12 | 0.161 | 0.007 |
| Pre-EC | Late | CSER low β | 12 | 0.112 | 0.014 |
| Pre-EC | Late | CSER high β | 12 | 0.112 | 0.029 |
| Pre-EC | Late | CSER low γ | 12 | 0.078 | 0.06 |
| Pre-EC | Late | CSER high γ | 12 | -0.087 | 0.119 |
| Pre-EO | Early | PSD δ | 18 | 0.724 | 0.272 |
| Pre-EO | Early | PSD θ | 18 | 0.657 | 0.286 |
| Pre-EO | Early | PSD α | 18 | 0.767 | 0.317 |
| Pre-EO | Early | PSD low β | 18 | 0.422 | 0.142 |
| Pre-EO | Early | PSD high β | 18 | 0.279 | 0.098 |
| Pre-EO | Early | PSD low γ | 18 | 0.173 | 0.057 |
| Pre-EO | Early | PSD high γ | 18 | 0.103 | 0.04 |
| Pre-EO | Early | Broadband | 18 | 0.263 | 0.083 |
| Pre-EO | Early | δ Oscillatory | 18 | 0.113 | 0.189 |
| Pre-EO | Early | δ Fractal | 18 | 0.55 | 0.553 |
| Pre-EO | Early | θ Oscillatory | 18 | 0.111 | 0.239 |
| Pre-EO | Early | θ Fractal | 18 | 0.55 | 0.553 |
| Pre-EO | Early | α Oscillatory | 18 | 0.545 | 0.483 |
| Pre-EO | Early | α Fractal | 18 | 0.292 | 0.186 |
| Pre-EO | Early | low β Oscillatory | 18 | 0.03 | 0.05 |
| Pre-EO | Early | low β Fractal | 18 | 0.181 | 0.102 |
| Pre-EO | Early | high β Oscillatory | 18 | 0.03 | 0.041 |
| Pre-EO | Early | high β Fractal | 18 | 0.068 | 0.037 |
| Pre-EO | Early | low γ Oscillatory | 18 | 0.005 | 0.008 |
| Pre-EO | Early | low γ Fractal | 18 | 0.031 | 0.018 |
| Pre-EO | Early | high γ Oscillatory | 18 | 0.013 | 0.011 |
| Pre-EO | Early | high γ Fractal | 18 | 0.002 | 0.002 |
| Pre-EO | Early | Broadband Oscillatory | 18 | 0.056 | 0.037 |
| Pre-EO | Early | Broadband Fractal | 18 | 0.095 | 0.059 |
| Pre-EO | Early | LZC | 18 | 0.407 | 0.053 |
| Pre-EO | Early | PLE 1-80hz | 18 | 0.11 | 0.009 |
| Pre-EO | Early | PLE 1-20hz | 18 | 0.086 | 0.029 |
| Pre-EO | Early | PLE 20-30hz | 18 | 0.08 | 0.02 |
| Pre-EO | Early | PLE 20-80hz | 18 | 0.115 | 0.01 |
| Pre-EO | Early | CSER Broadband | 18 | 0.74 | 0.188 |
| Pre-EO | Early | CSER δ | 18 | 0.076 | 0.007 |
| Pre-EO | Early | CSER θ | 18 | 0.096 | 0.01 |
| Pre-EO | Early | CSER α | 18 | 0.151 | 0.012 |
| Pre-EO | Early | CSER low β | 18 | 0.115 | 0.013 |
| Pre-EO | Early | CSER high β | 18 | 0.139 | 0.033 |
| Pre-EO | Early | CSER low γ | 18 | 0.136 | 0.055 |
| Pre-EO | Early | CSER high γ | 18 | 0.007 | 0.107 |
| Pre-EO | Late | PSD δ | 12 | 0.646 | 0.197 |
| Pre-EO | Late | PSD θ | 12 | 0.521 | 0.176 |
| Pre-EO | Late | PSD α | 12 | 0.646 | 0.19 |
| Pre-EO | Late | PSD low β | 12 | 0.419 | 0.132 |
| Pre-EO | Late | PSD high β | 12 | 0.26 | 0.064 |
| Pre-EO | Late | PSD low γ | 12 | 0.172 | 0.042 |
| Pre-EO | Late | PSD high γ | 12 | 0.102 | 0.027 |
| Pre-EO | Late | Broadband | 12 | 0.243 | 0.047 |
| Pre-EO | Late | δ Oscillatory | 12 | 0.085 | 0.067 |
| Pre-EO | Late | δ Fractal | 12 | 0.397 | 0.229 |
| Pre-EO | Late | θ Oscillatory | 12 | 0.015 | 0.051 |
| Pre-EO | Late | θ Fractal | 12 | 0.397 | 0.229 |
| Pre-EO | Late | α Oscillatory | 12 | 0.283 | 0.181 |
| Pre-EO | Late | α Fractal | 12 | 0.219 | 0.114 |
| Pre-EO | Late | low β Oscillatory | 12 | 0.059 | 0.067 |
| Pre-EO | Late | low β Fractal | 12 | 0.146 | 0.073 |
| Pre-EO | Late | high β Oscillatory | 12 | 0.022 | 0.022 |
| Pre-EO | Late | high β Fractal | 12 | 0.065 | 0.033 |
| Pre-EO | Late | low γ Oscillatory | 12 | 0.006 | 0.006 |
| Pre-EO | Late | low γ Fractal | 12 | 0.03 | 0.015 |
| Pre-EO | Late | high γ Oscillatory | 12 | 0.012 | 0.007 |
| Pre-EO | Late | high γ Fractal | 12 | 0.002 | 0.001 |
| Pre-EO | Late | Broadband Oscillatory | 12 | 0.035 | 0.017 |
| Pre-EO | Late | Broadband Fractal | 12 | 0.074 | 0.03 |
| Pre-EO | Late | LZC | 12 | 0.429 | 0.056 |
| Pre-EO | Late | PLE 1-80hz | 12 | 0.108 | 0.009 |
| Pre-EO | Late | PLE 1-20hz | 12 | 0.074 | 0.034 |
| Pre-EO | Late | PLE 20-30hz | 12 | 0.08 | 0.018 |
| Pre-EO | Late | PLE 20-80hz | 12 | 0.114 | 0.008 |
| Pre-EO | Late | CSER Broadband | 12 | 0.832 | 0.19 |
| Pre-EO | Late | CSER δ | 12 | 0.077 | 0.007 |
| Pre-EO | Late | CSER θ | 12 | 0.093 | 0.008 |
| Pre-EO | Late | CSER α | 12 | 0.154 | 0.01 |
| Pre-EO | Late | CSER low β | 12 | 0.124 | 0.014 |
| Pre-EO | Late | CSER high β | 12 | 0.147 | 0.025 |
| Pre-EO | Late | CSER low γ | 12 | 0.162 | 0.059 |
| Pre-EO | Late | CSER high γ | 12 | 0.05 | 0.11 |

Table S17: Baseline summary statistics of Early and Late responders average EEG metrics.

| **Comparison** | **Metric** | **p** |
| --- | --- | --- |
| Pre-EO | PSD δ | 0.369 |
| Pre-EO | PSD θ | 0.117 |
| Pre-EO | PSD α | 0.205 |
| Pre-EO | PSD low β | 0.950 |
| Pre-EO | PSD high β | 0.535 |
| Pre-EO | PSD low γ | 0.953 |
| Pre-EO | PSD high γ | 0.917 |
| Pre-EO | Broadband | 0.392 |
| Pre-EO | δ Oscillatory | 0.575 |
| Pre-EO | δ Fractal | 0.303 |
| Pre-EO | θ Oscillatory | 0.115 |
| Pre-EO | θ Fractal | 0.303 |
| Pre-EO | α Oscillatory | 0.047 |
| Pre-EO | α Fractal | 0.196 |
| Pre-EO | low β Oscillatory | 0.221 |
| Pre-EO | low β Fractal | 0.277 |
| Pre-EO | high β Oscillatory | 0.487 |
| Pre-EO | high β Fractal | 0.813 |
| Pre-EO | low γ Oscillatory | 0.890 |
| Pre-EO | low γ Fractal | 0.891 |
| Pre-EO | high γ Oscillatory | 0.852 |
| Pre-EO | high γ Fractal | 0.855 |
| Pre-EO | Broadband Oscillatory | 0.048 |
| Pre-EO | Broadband Fractal | 0.218 |
| Pre-EO | LZC | 0.300 |
| Pre-EO | PLE 1-80hz | 0.575 |
| Pre-EO | PLE 1-20hz | 0.324 |
| Pre-EO | PLE 20-30hz | 0.956 |
| Pre-EO | PLE 20-80hz | 0.908 |
| Pre-EO | CSER Broadband | 0.202 |
| Pre-EO | CSER δ | 0.946 |
| Pre-EO | CSER θ | 0.311 |
| Pre-EO | CSER α | 0.428 |
| Pre-EO | CSER low β | 0.091 |
| Pre-EO | CSER high β | 0.472 |
| Pre-EO | CSER low γ | 0.235 |
| Pre-EO | CSER high γ | 0.306 |
| Pre-EC | PSD δ | 0.378 |
| Pre-EC | PSD θ | 0.266 |
| Pre-EC | PSD α | 0.843 |
| Pre-EC | PSD low β | 0.864 |
| Pre-EC | PSD high β | 0.241 |
| Pre-EC | PSD low γ | 0.184 |
| Pre-EC | PSD high γ | 0.201 |
| Pre-EC | Broadband | 0.309 |
| Pre-EC | δ Oscillatory | 0.559 |
| Pre-EC | δ Fractal | 0.315 |
| Pre-EC | θ Oscillatory | 0.204 |
| Pre-EC | θ Fractal | 0.315 |
| Pre-EC | α Oscillatory | 0.496 |
| Pre-EC | α Fractal | 0.428 |
| Pre-EC | low β Oscillatory | 0.365 |
| Pre-EC | low β Fractal | 0.436 |
| Pre-EC | high β Oscillatory | 0.360 |
| Pre-EC | high β Fractal | 0.330 |
| Pre-EC | low γ Oscillatory | 0.195 |
| Pre-EC | low γ Fractal | 0.140 |
| Pre-EC | high γ Oscillatory | 0.178 |
| Pre-EC | high γ Fractal | 0.163 |
| Pre-EC | Broadband Oscillatory | 0.297 |
| Pre-EC | Broadband Fractal | 0.288 |
| Pre-EC | LZC | 0.572 |
| Pre-EC | PLE 1-80hz | 0.545 |
| Pre-EC | PLE 1-20hz | 0.625 |
| Pre-EC | PLE 20-30hz | 0.197 |
| Pre-EC | PLE 20-80hz | 0.580 |
| Pre-EC | CSER Broadband | 0.712 |
| Pre-EC | CSER δ | 0.747 |
| Pre-EC | CSER θ | 0.514 |
| Pre-EC | CSER α | 0.025 |
| Pre-EC | CSER low β | 0.289 |
| Pre-EC | CSER high β | 0.761 |
| Pre-EC | CSER low γ | 0.651 |
| Pre-EC | CSER high γ | 0.606 |

Table S18: Difference in EEG metrics between Early and Late responders. *Unpaired two-sided T-test with α-level = 0.05.

| **Comparison** | **EEG metrics** | **B coefficient** | **p value** | **p value (dose corrected)** |
| --- | --- | --- | --- | --- |
| Post-EO vs Pre-EO | PSD δ | - 0.08 | 0.210 | 0.210 |
| Post-EO vs Pre-EO | PSD θ | - 0.07 | 0.381 | 0.381 |
| Post-EO vs Pre-EO | PSD α | 0.01 | 0.918 | 0.918 |
| Post-EO vs Pre-EO | PSD low β | 0.05 | 0.190 | 0.190 |
| Post-EO vs Pre-EO | PSD high β | 0.01 | 0.716 | 0.716 |
| Post-EO vs Pre-EO | PSD low γ | -0.01 | 0.810 | 0.811 |
| Post-EO vs Pre-EO | PSD high γ | -0.01 | 0.664 | 0.664 |
| Post-EO vs Pre-EO | PSD broadband | - 0.004 | 0.869 | 0.869 |
| Post-EC vs Pre-EC | PSD δ | - 0.04 | 0.528 | 0.528 |
| Post-EC vs Pre-EC | PSD θ | - 0.03 | 0.769 | 0.769 |
| Post-EC vs Pre-EC | PSD α | 0.16 | 0.129 | 0.129 |
| Post-EC vs Pre-EC | PSD low β | 0.07 | 0.144 | 0.144 |
| Post-EC vs Pre-EC | PSD high β | 0.03 | 0.448 | 0.448 |
| Post-EC vs Pre-EC | PSD low γ | 0.004 | 0.900 | 0.900 |
| Post-EC vs Pre-EC | PSD high γ | < 0.001 | 0.994 | 0.994 |
| Post-EC vs Pre-EC | PSD broadband | 0.02 | 0.610 | 0.610 |
| Post-EO vs Pre-EO | Oscillatory δ | -0.07 | 0.224 | 0.224 |
| Post-EO vs Pre-EO | Oscillatory θ | -0.09 | 0.195 | 0.195 |
| Post-EO vs Pre-EO | Oscillatory α | -0.15 | 0.260 | 0.260 |
| Post-EO vs Pre-EO | Oscillatory low β | 0.04 | 0.082 | 0.082 |
| Post-EO vs Pre-EO | Oscillatory high β | 0.01 | 0.616 | 0.616 |
| Post-EO vs Pre-EO | Oscillatory low γ | - 0.001 | 0.835 | 0.835 |
| Post-EO vs Pre-EO | Oscillatory high γ | - 0.003 | 0.614 | 0.614 |
| Post-EO vs Pre-EO | Oscillatory broadband | - 0.01 | 0.239 | 0.239 |
| Post-EC vs Pre-EC | Oscillatory δ | 0.03 | 0.394 | 0.394 |
| Post-EC vs Pre-EC | Oscillatory θ | -0.17 | 0.230 | 0.230 |
| Post-EC vs Pre-EC | Oscillatory α | 0.12 | 0.648 | 0.648 |
| Post-EC vs Pre-EC | Oscillatory low β | 0.03 | 0.242 | 0.242 |
| Post-EC vs Pre-EC | Oscillatory high β | 0.02 | 0.181 | 0.181 |
| Post-EC vs Pre-EC | Oscillatory low γ | 0.01 | 0.543 | 0.543 |
| Post-EC vs Pre-EC | Oscillatory high γ | 0.01 | 0.559 | 0.559 |
| Post-EC vs Pre-EC | Oscillatory broadband | 0.01 | 0.589 | 0.589 |
| Post-EO vs Pre-EO | LZC (1-80hz; 500 Hz) | - 0.01 | 0.441 | 0.441 |
| Post-EC vs Pre-EC | LZC (1-80hz; 500 Hz) | - 0.04 | 0.025* | 0.025* |
| Post-EO vs Pre-EO | PLE_β_ | 0.01 | 0.077 | 0.077 |
| Post-EC vs Pre-EC | PLE_β_ | 0.02 | 0.019* | 0.019* |
| Post-EO vs Pre-EO | CSER δ | -0.002 | 0.406 | 0.406 |
| Post-EO vs Pre-EO | CSER θ | < 0.001 | 0.975 | 0.975 |
| Post-EO vs Pre-EO | CSER α | 0.015 | 0.003* | 0.003* |
| Post-EO vs Pre-EO | CSER low β | 0.01 | 0.005* | 0.005* |
| Post-EO vs Pre-EO | CSER high β | 0.003 | 0.724 | 0.724 |
| Post-EO vs Pre-EO | CSER low γ | - 0.01 | 0.682 | 0.682 |
| Post-EO vs Pre-EO | CSER high γ | - 0.01 | 0.790 | 0.790 |
| Post-EO vs Pre-EO | CSER broadband | 0.01 | 0.855 | 0.855 |
| Post-EC vs Pre-EC | CSER δ | - 0.01 | 0.061 | 0.061 |
| Post-EC vs Pre-EC | CSER θ | - 0.001 | 0.715 | 0.715 |
| Post-EC vs Pre-EC | CSER α | 0.02 | 0.004* | 0.004* |
| Post-EC vs Pre-EC | CSER low β | < 0.001 | 0.121 | 0.121 |
| Post-EC vs Pre-EC | CSER high β | - 0.01 | 0.285 | 0.285 |
| Post-EC vs Pre-EC | CSER low γ | - 0.04 | 0.050* | 0.050* |
| Post-EC vs Pre-EC | CSER high γ | -0.07 | 0.078 | 0.078 |
| Post-EC vs Pre-EC | CSER broadband | -0.12 | 0.100 | 0.100 |

Table S19: Difference in EEG metrics pre- to post-ketamine between Early vs Late responders. *Linear mixed-effect model with α-level = 0.05.

References:

1. Wen H, Liu Z (2016): Separating Fractal and Oscillatory Components in the Power Spectrum of Neurophysiological Signal. *Brain Topogr* 29: 13–26.

2. Muthukumaraswamy SD, Liley DTJ (2018): 1/f electrophysiological spectra in resting and drug-induced states can be explained by the dynamics of multiple oscillatory relaxation processes. *NeuroImage* 179: 582–595.

3. Kaspar F, Schuster HG (1987): Easily calculable measure for the complexity of spatiotemporal patterns. *Phys Rev A* 36: 842–848.

4. Mediano PAM, Rosas FE, Luppi AI, Noreika V, Seth AK, Carhart-Harris RL, *et al.* (2023): Spectrally and temporally resolved estimation of neural signal diversity. *eLife* 12. https://doi.org/10.7554/eLife.88683

5. Maris E, Oostenveld R (2007): Nonparametric statistical testing of EEG- and MEG-data. *J Neurosci Methods* 164: 177–190.

6. Tukey JW (1977): Some thoughts on clinical trials, especially problems of multiplicity. *Science* 198: 679–684.

7. Benjamini Y, Hochberg Y (1995): Controlling the False Discovery Rate: A Practical and Powerful Approach to Multiple Testing. *J R Stat Soc Ser B Methodol* 57: 289–300.
